# Mean Reversion and Heavy Tails: Characterizing Time-Series Data Using Ornstein–Uhlenbeck Processes and Machine Learning

**DOI:** 10.3390/s26041263

**Published:** 2026-02-14

**Authors:** Sebastian Raubitzek, Sebastian Schrittwieser, Georg Goldenits, Alexander Schatten, Kevin Mallinger

**Affiliations:** 1 Complexity and Resilience Research Group, SBA Research gGmbH, Floragasse 7/5.OG, 1040 Vienna, Austria; sraubitzek2@sba-research.org (S.R.); ggoldenits@sba-research.org (G.G.); aschatten@sba-research.org (A.S.); kmallinger@sba-research.org (K.M.); 2 Christian Doppler Laboratory for Assurance and Transparency in Software Protection, Faculty of Computer Science, University of Vienna, Kolingasse 14-16, 1090 Vienna, Austria

**Keywords:** Ornstein–Uhlenbeck, Lévy, Gaussian, mean reversion, complexity metrics, machine learning

## Abstract

We present a supervised learning method to estimate two local descriptors of time-series dynamics, the mean-reversion rate θ and a heavy-tail estimate α, from short windows of data. These parameters summarize recovery behavior and tail heaviness and are useful for interpreting stochastic signals in sensing applications. The method is trained on synthetic, dimensionless Ornstein–Uhlenbeck processes with α-stable noise, ensuring robustness for non-Gaussian and heavy-tailed inputs. Gradient-boosted tree models (CatBoost) map window-level statistical features to discrete α and θ categories with high accuracy and predominantly adjacent-class confusion. Using the same trained models, we analyze daily financial returns, daily sunspot numbers, and NASA POWER climate fields for Austria. The method detects changes in local dynamics, including shifts in the financial tail structure after 2010, weaker and more irregular solar cycles after 2005, and a redistribution in clear-sky shortwave irradiance around 2000. Because it relies only on short windows and requires no domain-specific tuning, the framework provides a compact diagnostic tool for signal processing, supporting the characterization of local variability, detection of regime changes, and decision making in settings where long-term stationarity is not guaranteed.

## 1. Introduction

Mean reversion and heavy tails are two recurring features of real-world time series. The Ornstein–Uhlenbeck (OU) process captures mean reversion through a linear drift toward equilibrium [1]. In its Gaussian form, increments are light-tailed, and the second-order structure fully characterizes variability. Many applications, however, show departures from Gaussianity: returns in finance, intermittency in environmental series, and burst-like activity in solar and geophysical records [2,3]. A tractable extension replaces Brownian motion by a symmetric α-stable Lévy driver with stability index α∈(0,2], which preserves mean reversion while allowing heavy tails and occasional large jumps [4,5]. When α<2, the variance is infinite, so methods relying on low-order moments can become unstable or biased.

Jointly estimating a mean-reversion rate θ and a tail index α from finite, noisy data is difficult. Likelihood-based inference for stable-driven OU models requires either long samples or numerical inversion of characteristic functions and careful treatment of normalization; performance deteriorates as α moves away from the Gaussian boundary [6]. Moment and cumulant approaches are not well defined when moments diverge. Tail-quantile and peak-over-threshold methods focus on extremes but ignore the temporal structure that informs the mean reversion rate θ. These gaps motivate an approach that uses short-window information beyond low-order moments that scales to diverse types of data/signals.

**Why is this important?** Many operational decisions depend on distinguishing transient noise from persistent regime changes. In finance, separating Gaussian from heavy-tailed periods and identifying changes in reversion speed affects risk limits, hedging, and stress testing [2,7,8,9,10,11]. Ref. [9] provides an early and systematic analysis of mean reversion in financial time series based on variance-ratio methods. Their results demonstrate that deviations from a random-walk benchmark can be detected in short windows and that mean-reverting dynamics appear intermittently across asset classes. This aligns with our objective of classifying local windows by reversion strength rather than assuming stable long-run parameters. Relatedly, ref. [12] shows a structural increase in complexity and a persistent decline in out-of-sample predictability for major equity indices. The work shows that increases in approximate and sample entropy correspond to weaker forecasting performance in a wide range of Machine Learning (ML) models. This supports our motivation to jointly screen windows for Gaussian vs. non-Gaussian features and for strong vs. weak reversion, since both characteristics are linked to shifts in local predictability and model stability. In environmental monitoring, heavy-tailed windows and weak short-horizon reversion indicate intermittent events and regime persistence, which influence planning and early warning [13,14,15]. In scientific data analysis, reliable identification of heavy tails and mean reversion supports model selection and hypothesis testing when moments are undefined or uninformative [4,16]. A window-based classifier that does not rely on strong distributional assumptions enables rolling-window-based analysis, cross-domain comparability, and robust summaries in settings where classical estimators fail, are expensive to deploy, or require long observation horizons, especially for live data stream analysis [17,18,19].

We address this by developing a supervised learning approach that maps window-level features to discrete classes for θ and α. Each sample is a fixed-length segment of a dimensionless OU process; a vector of numeric descriptors summarizes ordinal patterns and short-horizon dynamics without assuming finite variance (e.g., permutation-based features [19]). We use CatBoost, a gradient-boosted decision-tree method designed for tabular inputs [20,21]. CatBoost’s ordered boosting and oblivious-tree structure provide stable and fast training and work well with categorical, as well as in our case, purely numeric features.

The novelty of this approach lies in coupling exact, stable-driven OU simulations with window-level supervised learning to obtain calibrated, discrete regime labels at horizons where classical estimators are unreliable. Unlike traditional inference pipelines, the method directly targets operational window sizes and preserves interpretability through explicit links to established stochastic parameters.

**Figure 1:**
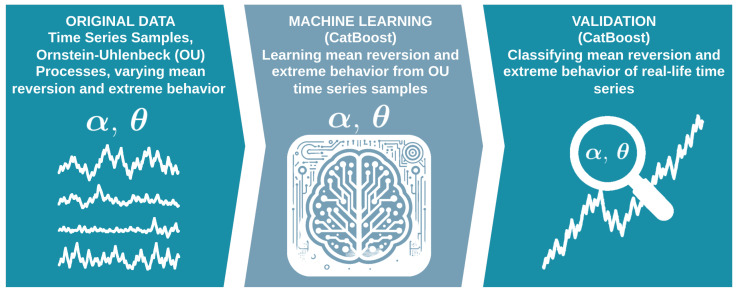
Overview of our approach, from left to right: Left: Synthetic time series are generated from Ornstein–Uhlenbeck processes with varying mean-reversion strength θ and tail behavior α to cover a wide range of Gaussian and non-Gaussian regimes. Center: A supervised learning model (CatBoost) is trained on these samples to learn how mean reversion and extreme-value behavior are expressed in short time windows. Right: The trained classifier is applied to real-world financial time series to validate the approach. The validation step shows how the method distinguishes windows with strong vs. weak reversion and Gaussian vs. heavy-tailed behavior in real-life data, e.g., financial and environmental data.

The synthetic data, i.e., the employed Ornstein–Uhlenbeck processes, establish the ground truth and control the task’s granularity. We generate dimensionless OU series on a grid of α∈{0.05,0.5,1.0,1.5,2.0} and $\theta \in \{10^{-6},2,4,8,16,32\}$ using exact discrete transitions for both Gaussian and stable drivers. Base series have fixed lengths and are segmented into overlapping windows of sizes w∈{50,100,250,365} with shift w/2. For every *w*, we compute the same feature set, train two independent CatBoost classifiers (one for α and one for θ), and evaluate on independent-seed test sets that share the parameter grids but not the noise realizations. We report per-class precision, recall, and $F_{1}$ score, overall accuracy, and weighted $F_{1}$ score, along with absolute and row-normalized confusion matrices. This approach quantifies (i) separability as a function of window length and (ii) where misclassifications occur in parameter space. To reflect a frequent screening use case, we also evaluate the same trained models on binary classifications as well, i.e., Gaussian vs. non-Gaussian (α) and mean reversion vs. no mean reversion (θ)—without retraining. Finally, we test, evaluate, and interpret our approach on several real-life time series. Figure 1 depicts the main idea.

A central objective of this work is to demonstrate that short-window statistical diagnostics can recover meaningful regime information across real-world systems. We therefore analyze heterogeneous datasets—daily financial returns, daily sunspot numbers, and environmental measurements—not because they share a physical mechanism but because they offer complementary testbeds with a well-documented regime structure. First, these domains exhibit transitions between markedly different dynamical states (e.g., volatility shifts in financial markets and solar cycle phases in sunspot activity), making them suitable for evaluating whether short-window classifiers can detect changes that traditionally require long-term aggregation [2,22]. Second, each dataset provides a distinct stress case for robustness: financial returns contain heavy tails and clustered volatility, sunspot numbers have quasi-periodic but irregular cycles, and NASA POWER data include meteorological noise and intermittent extremes. Demonstrating consistent performance across such systems highlights that the proposed framework functions as a general-purpose analysis tool.

More broadly, the machine learning models introduced here should be understood as methodological tools rather than domain-specific predictors. Many classical approaches to estimating distributional properties or mean-reversion rates rely on long samples and strong parametric assumptions; this limits their usefulness when regime changes occur on short horizons or when assumptions (such as finite variance) do not hold. The presented machine learning approach, by contrast, enables data-driven detection of local dynamical structures from relatively small windows, offering a scalable approach for identifying short-term regime changes in settings where traditional estimators fail or require prohibitively long data records.

Further, we compare the classifier-based results to established complexity metrics and classical estimators to clarify what information is gained or lost at short horizons. Classical heavy-tail estimators and mean-reversion measures are well understood asymptotically, but their finite-sample behavior on short windows can be unstable or poorly calibrated. By contrasting their window-wise correlations with α and θ against the supervised predictions, we provide a reference point for interpreting the machine learning outputs and for understanding which aspects of the signal are already captured by traditional diagnostics and how well these traditional diagnostics map to our α and θ ground truth.

This article makes five contributions. First, it provides an exact, dimensionless simulation of stable-driven OU paths across a wide (θ,α) grid for supervised learning. Second, it shows that window-level features paired with boosted tree models recover both the tail index and reversion rate with useful fidelity, with errors concentrated between neighboring classes. Third, it documents a compact binary analysis that reuses the same models to flag Gaussian vs. non-Gaussian regimes and the presence vs. absence of short-horizon mean reversion. Fourth, it compares our developed machine learning models to traditional extreme value, heavy-tail, and mean-reversion diagnostics. Fifth, it applies the method to financial, environmental, and solar time series to illustrate how regime mixes shift across assets, variables, and time intervals.

The remainder of this paper is organized as follows. Section 3 introduces the Ornstein–Uhlenbeck (OU) model with α-stable increments, the exact discretization used for generating synthetic benchmarks, and the window-level feature set. Section 4 specifies the supervised learning setup, including the CatBoost classifiers, the windowing procedure, data splits, and the training protocol. Section 5 outlines the validation framework and reports the full multi-class results on synthetic data together with the corresponding confusion matrices; the complementary binary evaluations are provided in Appendix A. Section 6 follows the initial validation and compares the classifier outputs to established complexity metrics and classical estimators, assessing window-wise correlations for the known ground truth. Section 7, Section 8 and Section 9 present the empirical applications to financial returns, daily sunspot numbers, and NASA POWER climate variables for Austria, illustrating how the method identifies short-window regime changes across heterogeneous real-world signals. Section 10 summarizes the main findings and discusses limitations and practical use cases. Section 11 concludes this article.

## 2. Related Work

A growing line of research connects time-series *complexity* with the achievable accuracy of machine and deep learning predictors. Survey evidence shows that entropy- and fractal-based descriptors (e.g., Hurst exponent, fractal dimension, Lyapunov spectrum) help quantify persistence, intermittency, and predictability, and that models which account for these properties tend to perform better on difficult, noisy signals [23]. Building on this, several studies operationalize the link by filtering or shaping model outputs according to signal complexity: ensemble LSTMs combined with complexity filters reduce error and limit overfitting without expensive hyperparameter tuning [24]; reconstructed phase spaces and phase-space–consistent filtering stabilize autoregressive neural forecasts across datasets [25]; and stochastic interpolation that preserves attractor geometry (via fractional Brownian bridges) improves data density while maintaining the dynamical structure [26]. Complementary work shows that complexity-aware interpolation—especially when guided by fractal metrics—can improve LSTM performance on short or coarse data and better handle long-memory behavior [27].

Our contribution follows this arc but targets the *interpretable* regime classification that is useful beyond prediction alone. Instead of using complexity solely as auxiliary features, we estimate two window-local, physically meaningful quantities: tail heaviness (α) and mean-reversion speed (θ). Trained on OU processes with α-stable increments, the resulting classifiers act as compact “regime sensors” that travel across domains. We validate them on synthetic data and then apply them to three heterogeneous real datasets (finance, sunspots, and environmental series), showing that the same complexity-informed framing supports both predictive tasks, where prior work emphasized LSTM ensembles and interpolation [24,25,26,27,28], and diagnostic task, where our α and θ labels summarize distributional shape and recovery dynamics. In short, this extends the complexity–predictability link surveyed by Raubitzek and Neubauer [23] into a regime-mapping tool that is practical for out-of-sample monitoring in real systems.

## 3. Ornstein–Uhlenbeck Processes

The Ornstein–Uhlenbeck (OU) process was introduced by Uhlenbeck and Ornstein to model velocity relaxation in a gas [1]. It is now a standard mean-reverting stochastic differential equation (SDE) with broad use in physics, finance, and engineering [29,30,31]. In its basic form, the process $\left\{X_{t}:t\geq 0\right\}$ satisfies

(1) $$dX_{t}\;=\;-\theta \;\left(X_{t}-\mu \right)\;dt\;+\;\sigma \;dW_{t},$$

where *t* denotes time, θ>0 is the mean-reversion rate towards the level μ∈R, σ>0 is the diffusion (noise) scale, and ${\left\{W_{t}\right\}}_{t\geq 0}$ is a standard Brownian motion. We write N(m,v) for the normal distribution with mean *m* and variance *v*. Equation (1) admits the explicit solution obtained by variation of constants,

(2) $$X_{t}\;=\;\mu +\left(X_{0}-\mu \right)\;e^{-\theta t}+\sigma \int_{0}^{t}e^{-\theta (t-s)}\;dW_{s},$$

where $X_{0}$ is the initial value at t=0. Conditioned on $X_{0}=x_{0}$, the process is Gaussian with

(3) $$E\left[X_{t}|X_{0}=x_{0}\right]\;=\;\mu +\left(x_{0}-\mu \right)e^{-\theta t},\;\mathrm{Var}\left(X_{t}|X_{0}=x_{0}\right)\;=\;\frac{\sigma^{2}}{2\theta }\left(1-e^{-2\theta t}\right).$$

As t→∞, the conditional mean converges to μ and the conditional variance converges to $\sigma^{2}/\left(2\theta \right)$, hence

(4) $$X_{t}\Rightarrow N\;\left(\mu ,\frac{\sigma^{2}}{2\theta }\right).$$

If the process is initialized from the stationary distribution (or after transients are negligible), the stationary autocovariance is

(5) $$\mathrm{Cov}\left(X_{t},X_{t+\tau }\right)\;=\;\frac{\sigma^{2}}{2\theta }\;e^{-\theta \tau },\;\tau \geq 0,$$

so the characteristic relaxation time is 1/θ. This relaxation time quantifies the temporal scale over which correlations decay and the process loses memory of past states. Specifically, after a time interval of order 1/θ, the autocovariance decreases by a factor of $e^{-1}$, indicating that deviations from the mean level μ are largely dissipated. Larger values of θ therefore correspond to faster mean reversion and shorter memory, while smaller values of θ imply slower relaxation and more persistent temporal dependence. Over a fixed sampling interval Δt>0, the transition density is Gaussian. Sampling at times $t_{n}=n\Delta t$ and writing $X_{n}:=X_{t_{n}}$ gives the exact recursion

(6) $$X_{n+1}\;=\;\mu +\left(X_{n}-\mu \right)\;e^{-\theta \Delta t}+\sigma \;\sqrt{\frac{1-e^{-2\theta \Delta t}}{2\theta }}\;Z_{n},\;Z_{n}∼N\left(0,1\right)\;i.i.d.$$

Equivalently, the conditional distribution is

(7) $$X_{n+1}|X_{n}\;∼\;N\;\left(\mu +\left(X_{n}-\mu \right)e^{-\theta \Delta t},\;\frac{\sigma^{2}}{2\theta }\left(1-e^{-2\theta \Delta t}\right)\right).$$

This is the closed-form (non-Euler–Maruyama) update; therefore, no additional $\sqrt{\Delta t}$ factor multiplies $Z_{n}$. For parameter inference, it is convenient to remove location and scale while preserving mean reversion. Define the dimensionless, centered, and scaled process

(8) $$Y_{t}\;=\;\sqrt{\frac{2\theta }{\sigma^{2}}}\;\left(X_{t}-\mu \right).$$

Since $Y_{t}$ in Equation (8) is affine in $X_{t}$, Itô’s formula reduces to differentiation of the map $g\left(x\right)=\sqrt{2\theta /\sigma^{2}}\;\left(x-\mu \right)$ with $g^{\prime }\left(x\right)=\sqrt{2\theta /\sigma^{2}}$ and $g^{\prime \prime }\left(x\right)=0$:

(9) $$\begin{array}{cc} dY_{t} & =g^{\prime }\left(X_{t}\right)\;dX_{t}+\frac{1}{2}g^{\prime \prime }\left(X_{t}\right)\sigma^{2}\;dt\\ \; & =\sqrt{\frac{2\theta }{\sigma^{2}}}\left(-\theta \left(X_{t}-\mu \right)\;dt+\sigma \;dW_{t}\right)\\ \; & =-\theta \sqrt{\frac{2\theta }{\sigma^{2}}}\left(X_{t}-\mu \right)\;dt+\sqrt{2\theta }\;dW_{t}\\ \; & =-\theta \;Y_{t}\;dt+\sqrt{2\theta }\;dW_{t}. \end{array}$$

Hence, in the stationary regime, $Y_{t}$ has mean 0 and variance 1. The exact discrete transition in Equation (10) over Δt is

(10) $$Y_{n+1}\;=\;e^{-\theta \Delta t}\;Y_{n}\;+\;\sqrt{\;1-e^{-2\theta \Delta t}\;}\;Z_{n},\;Z_{n}∼N\left(0,1\right)\;i.i.d.$$

Equation (10) keeps θ as the single free parameter in the Gaussian OU case.

### 3.1. Replacing the Wiener Driver by Symmetric α-Stable Noise

Empirical series in several domains exhibit heavy tails and jumps that occur more often than Gaussian models predict. A natural extension replaces $dW_{t}$ in Equation (9) by the increment of a symmetric (β=0) α-stable Lévy process $L^{\left(\alpha \right)}$. A random variable *X* is *stable* if sums of i.i.d. copies are equal in law to a location–scale transform of *X*. We write X∼S(α,β,γ,δ) with stability α∈(0,2], skewness β∈[−1,1], scale γ>0, and location δ∈R. For α≠1, the characteristic function is

(11) $$\varphi_{X}\left(t\right)\;=\;\exp\;\left\{i\delta t\;-\;\gamma^{\alpha }\;{\left|t\right|}^{\alpha }\left[1-i\beta \;\mathrm{sgn}(t)\;\tan(\pi \alpha /2)\right]\right\}.$$

For α=1, the characteristic function has a different (logarithmic) form; in this work we use the symmetric case β=0, for which the α=1 characteristic function reduces to $\varphi_{X}\left(t\right)=\exp\left\{i\delta t-\gamma |t|\right\}$. Special cases: α=2 gives $N(\delta ,2\gamma^{2})$; with α=1 and β=0, one obtains the Cauchy law. Moments satisfy ${E|X|}^{p}<\infty$ if p<α. Let $\left\{L_{t}^{\left(\alpha \right)}:t\geq 0\right\}$ be a symmetric α-stable Lévy motion with independent, stationary increments and

$$L_{0}^{\left(\alpha \right)}=0,\;L_{t}^{\left(\alpha \right)}-L_{s}^{\left(\alpha \right)}\;∼\;S\;\left(\alpha ,\;0,\;{\left(t-s\right)}^{1/\alpha },\;0\right),\;0\leq s<t,$$

i.e., the increment scale grows like ${\left(t-s\right)}^{1/\alpha }$ (unit scale at t=1). We consider the Lévy-driven OU SDE

(12) $$dY_{t}\;=\;-\theta \;Y_{t}\;dt\;+\;\sigma_{\alpha }\;dL_{t}^{\left(\alpha \right)},\;\theta >0,\;0<\alpha \leq 2.$$

The solution admits the moving-average representation

(13) $$Y_{t}\;=\;e^{-\theta t}Y_{0}\;+\;\sigma_{\alpha }\int_{0}^{t}e^{-\theta (t-s)}\;dL_{s}^{\left(\alpha \right)}.$$

In the stationary regime (or for t→∞), the marginal distribution of $Y_{t}$ is again α-stable [32]. To determine its scale, use the stability property of stochastic integrals with respect to $L^{\left(\alpha \right)}$: for deterministic $f\in L^{\alpha }\left(R_{+}\right)$,

$$\int_{0}^{\infty }f\left(s\right)\;dL_{s}^{\left(\alpha \right)}\;∼\;S\;\left(\alpha ,0,{\left(\int_{0}^{\infty }{\left|f\left(s\right)\right|}^{\alpha }ds\right)}^{1/\alpha },0\right).$$

With $f\left(s\right)=\sigma_{\alpha }e^{-\theta s}$, we obtain the stationary scale parameter

(14) $$\gamma_{\infty }\;=\;\sigma_{\alpha }{\left(\int_{0}^{\infty }e^{-\alpha \theta s}\;ds\right)}^{1/\alpha }\;=\;\sigma_{\alpha }{\left(\frac{1}{\alpha \theta }\right)}^{1/\alpha }.$$

To keep the dimensionless convention (unit stationary *scale* for all α), we choose

(15) $$\sigma_{\alpha }\;=\;{\left(\alpha \theta \right)}^{1/\alpha },$$

which yields $\gamma_{\infty }=1$ and hence the stationary marginal S(α,0,1,0) for every α. Let Δt>0 and $t_{n}=n\Delta t$. From Equation (13),

$$Y_{t_{n+1}}=e^{-\theta \Delta t}Y_{t_{n}}+\sigma_{\alpha }\int_{t_{n}}^{t_{n+1}}e^{-\theta (t_{n+1}-s)}\;dL_{s}^{\left(\alpha \right)}.$$

By stationarity and independence of Lévy increments, the integral term is independent of $Y_{t_{n}}$ and has the same law as $\sigma_{\alpha }\int_{0}^{\Delta t}e^{-\theta (\Delta t-u)}\;dL_{u}^{\left(\alpha \right)}$. Using the scaling rule for stable integrals,

(16) $$\begin{array}{cc} \sigma_{\alpha }\int_{0}^{\Delta t}e^{-\theta (\Delta t-u)}\;dL_{u}^{\left(\alpha \right)}\; & \;∼\;S\;\left(\alpha ,0,\;\sigma_{\alpha }{\left(\int_{0}^{\Delta t}e^{-\alpha \theta (\Delta t-u)}\;du\right)}^{1/\alpha },\;0\right)\\ \; & \;=\;S\;\left(\alpha ,0,\;\sigma_{\alpha }{\left(\int_{0}^{\Delta t}e^{-\alpha \theta v}\;dv\right)}^{1/\alpha },\;0\right), \end{array}$$

where v=Δt−u. Therefore, the exact transition can be written as

(17) $$Y_{n+1}\;=\;e^{-\theta \Delta t}\;Y_{n}\;+\;\sigma_{\alpha }\;{\left(\int_{0}^{\Delta t}e^{-\alpha \theta v}\;dv\right)}^{1/\alpha }S_{n},\;S_{n}∼S\left(\alpha ,0,1,0\right)\;i.i.d.$$

Evaluating the integral gives $\int_{0}^{\Delta t}e^{-\alpha \theta v}\;dv=\left(1-e^{-\alpha \theta \Delta t}\right)/\left(\alpha \theta \right)$, so with the normalization Equation (15), this simplifies to

(18) $$Y_{n+1}\;=\;e^{-\theta \Delta t}\;Y_{n}\;+\;{\left(1-e^{-\alpha \theta \Delta t}\right)}^{1/\alpha }\;S_{n},\;S_{n}∼S\left(\alpha ,0,1,0\right)\;i.i.d.$$

For α=2, $S_{n}$ corresponds to a Gaussian draw and Equation (18) reduces to the Gaussian transition Equation (10). In the implementation, we draw $S_{n}$ using scipy.stats.levy_stable. At α=2, β=0, and scale=1, this sampler returns N(0,2) rather than N(0,1). Therefore, when α=2, we rescale the draw by $1/\sqrt{2}$ to match Z∼N(0,1) in (10). Alternatively, one may bypass the stable sampler at α=2 and draw *Z* directly. Specifically, the two cases are given by the following exact one-step updates:

(19) $$Y_{n+1}=e^{-\theta \Delta t}\;Y_{n}+\left\{\begin{array}{cc} {\left(1-e^{-\alpha \theta \Delta t}\right)}^{1/\alpha }\;S_{n},\; & \alpha \in \left(0,2\right),\;S_{n}∼S\left(\alpha ,0,1,0\right),\\ \sqrt{1-e^{-2\theta \Delta t}}\;Z_{n},\; & \alpha =2,\;Z_{n}∼N\left(0,1\right), \end{array}\right.$$

where all innovations are i.i.d. and independent of $Y_{n}$. With the following variables:

θ exponential pull toward zero; larger θ implies faster mean reversion.
α tail index; smaller α yields heavier tails and larger jumps; α=2 is Gaussian.
$\sigma_{\alpha }$ noise scale in Equation (12); with $\sigma_{\alpha }={\left(\alpha \theta \right)}^{1/\alpha }$, the stationary marginal scale is 1 for all α.

The combination of mean-reverting drift and heavy-tailed shocks provides a flexible baseline for time series where both features are present. See Samorodnitsky and Taqqu [4], Nolan [16], and Marquardt [32] for detailed background information.

### 3.2. Simulation Setup and Implementation

We use symmetric α-stable noise (β=0) with α∈{0.05,0.5,1.0,1.5,2.0}, spanning the range from Gaussian (α=2) to extremely heavy-tailed regimes (small α). Stable variates are generated with

(20) $$S_{n}\;∼\;\mathrm{levy}\_\mathrm{stable}.\mathrm{rvs}\left(\alpha ,\;\beta =0,\;\mathrm{scale}=1,\;\mathrm{loc}=0\right),$$

which returns i.i.d. draws from S(α,0,1,0). Inserting these into (18) yields the exact one-step update used for simulation. At α=2, scale=1 corresponds to N(0,2) (i.e., norm with scale$=\sqrt{2}$), so we rescale by $1/\sqrt{2}$ to obtain Z∼N(0,1) [33]. To generate time series for supervised learning, we discretize the *dimensionless* OU model on a uniform grid. We use the exact transition from Equations  (10) and (19):

(21) $$Y_{n+1}=e^{-\theta \;\Delta t}Y_{n}+\sqrt{1-e^{-2\;\theta \;\Delta t}}\;Z_{n},\;Z_{n}∼N\left(0,1\right)\;i.i.d.$$

For α∈(0,2], we use the exact transition in Equations (18) and (19):

(22) $$Y_{n+1}\;=\;e^{-\theta \;\Delta t}Y_{n}\;+\;{\left(1-e^{-\alpha \;\theta \;\Delta t}\right)}^{1/\alpha }S_{n},\;S_{n}∼S\left(\alpha ,0,1,0\right)\;i.i.d.$$

**Figure 2:**
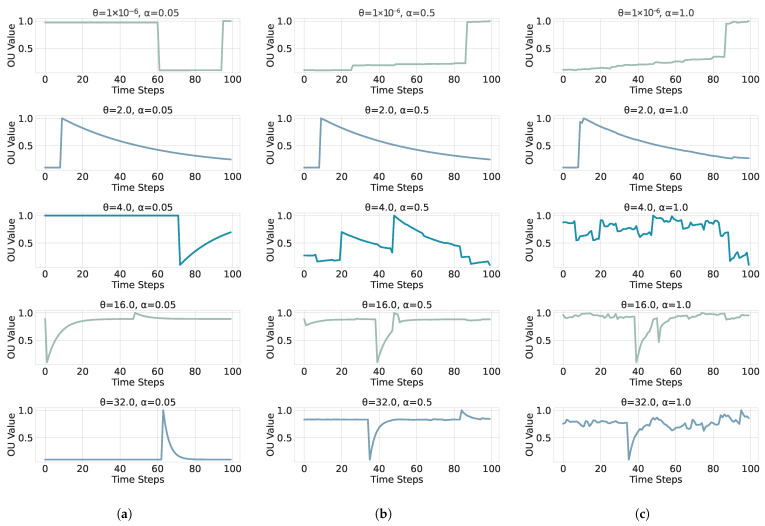
Dimensionless OU sample paths with symmetric α-stable innovations (β=0) for α∈{0.05,0.5,1.0} (panels (**a**–**c**)). Smaller α produces heavier tails and more frequent large jumps. The mean-reversion rate θ and the sampling step Δt are the same across panels.

**Figure 3:**
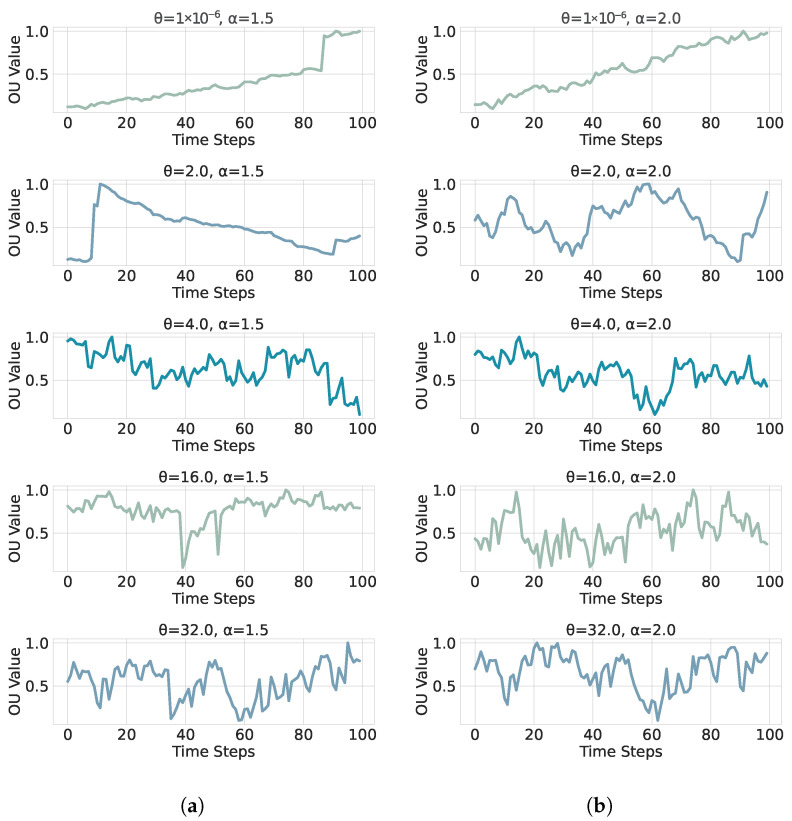
Dimensionless OU sample paths with symmetric α-stable innovations (β=0) for α∈{1.5,2.0} (panels (**a**,**b**)). The case α=2 corresponds to the Gaussian OU model. The mean-reversion rate θ and the sampling step Δt are the same across panels.

This choice is consistent with the SDE normalization $\sigma_{\alpha }={\left(\alpha \theta \right)}^{1/\alpha }$ and keeps the stationary marginal scale equal to one for all α. The resulting dimensionless series provide a homogeneous input space across (θ,α) for feature extraction and learning. These dimensionless Ornstein–Uhlenbeck processes are depicted in Figure 2 and Figure 3 for several parameter combinations (θ,α). We explore five values for the stability index α:

(23) α∈{0.05,0.5,1.0,1.5,2.0},

each with skewness fixed at β=0 (symmetric law). The choice spans the full range from the Gaussian case (α=2) to an extreme heavy-tail regime (α=0.05). For reference:

(1) α=2.0: normal distribution; finite mean and variance.
(2) α=1.5: infinite variance but finite mean; moderate tails.
(3) α=1.0: Cauchy-type; mean and variance both infinite.
(4) α=0.5: very heavy tails; large jumps common.
(5) α=0.05: extreme heavy-tail regime with rare but dominating outliers.

We define a separate grid for the mean-reversion rate θ:

(24) $$\theta \in \in \{10^{-6},2,4,8,16,32\}\;,$$

here $\theta_{j}>0$, controls how fast the process returns to equilibrium. We further keep the stationary marginal scale equal to one by setting $\sigma_{\alpha }={\left(\alpha \theta \right)}^{1/\alpha }$, see Equation (15). This normalization allows a direct comparison of paths across different α.

Stable increments are generated with scipy.stats.levy_stable.rvs(α,β=0, scale=1,loc=0) [33,34]. The call returns i.i.d. variables $S_{n}∼S\left(\alpha ,0,1,0\right)$ that feed the discrete update below. When α=2, SciPy’s stable law corresponds to norm with scale$=\sqrt{2}$ [34]; therefore, we rescale by $1/\sqrt{2}$ when we require Z∼N(0,1).

Let $Y_{n}=Y_{t_{n}}$ with $t_{n}=n\Delta t$. The exact transition of Equation (12) over one step Δt is

(25) $$Y_{n+1}\;=\;e^{-\theta \Delta t}\;Y_{n}\;+\;{\left(1-e^{-\alpha \theta \Delta t}\right)}^{1/\alpha }\;S_{n},\;S_{n}∼S\left(\alpha ,0,1,0\right)\;i.i.d.$$

*Derivation.* Start from the moving-average solution $Y_{t_{n+1}}=e^{-\theta \Delta t}Y_{t_{n}}+\sigma_{\alpha }\int_{t_{n}}^{t_{n+1}}e^{-\theta (t_{n+1}-s)}\;dL_{s}^{\left(\alpha \right)}$. By independent increments, the integral term is independent of $Y_{t_{n}}$ and has the same law as $\sigma_{\alpha }\int_{0}^{\Delta t}e^{-\theta (\Delta t-u)}\;dL_{u}^{\left(\alpha \right)}$. For symmetric α-stable Lévy motion, the scale of such an integral equals $\sigma_{\alpha }{\left(\int_{0}^{\Delta t}e^{-\alpha \theta v}\;dv\right)}^{1/\alpha }=\sigma_{\alpha }{\left(\frac{1-e^{-\alpha \theta \Delta t}}{\alpha \theta }\right)}^{1/\alpha }$, and with $\sigma_{\alpha }={\left(\alpha \theta \right)}^{1/\alpha }$, this becomes ${\left(1-e^{-\alpha \theta \Delta t}\right)}^{1/\alpha }$. For α=2, we obtain

(26) $$Y_{n+1}=e^{-\theta \Delta t}\;Y_{n}+\sqrt{1-e^{-2\theta \Delta t}}\;Z_{n},\;Z_{n}∼N\left(0,1\right)\;i.i.d.,$$

which, again, matches the exact Gaussian OU update, Equation (19). Again, defined by:  
$e^{-\theta \Delta t}$deterministic decay over one step.${\left(1-e^{-\alpha \theta \Delta t}\right)}^{1/\alpha }$innovation scale; small when Δt≪1/θ.$S_{n}$independent symmetric α-stable driver controlling tail heaviness via α.

Equation (25), respectively, Equation (19) is used throughout the study to simulate training and test paths for every (θ,α) pair. Our implementation uses scipy.stats.levy_stable (in Python 3.10.9) for the random process $S_{n}∼S\left(\alpha ,0,1,0\right)$. SciPy draws random numbers from a Lévy-stable law with the rvs method of the class scipy.stats.levy_stable [34]. Internally, this routine follows the Chambers–Mallows–Stuck (CMS) transformation [35].

Dimensionless scaling is critical for ML comparability because it removes the location and scale degrees of freedom and fixes a common stationary marginal scale across different (θ,α) settings. This ensures that learned features and decision boundaries reflect differences in mean reversion and tail behavior rather than trivial changes in units or amplitude. Using exact discrete transitions instead of Euler–Maruyama updates avoids discretization bias that is pronounced for short windows and coarse sampling, and it prevents artificial smoothing of jumps in the α-stable case. This is important for the following because our classifier is trained on window-level statistics, where small systematic simulation errors can translate into consistent label noise and thus degrade generalization. This, as we will later see, will still be present and visible by confusions for non-extreme regimes where α and θ are not at their respective boundary (highest/lowest) values.

## 4. Machine Learning

We use CatBoost, a gradient-boosting decision tree method, as the primary classifier for all window-level experiments [20,21]. Gradient boosting builds an ensemble sequentially, with each tree trained to reduce residual errors of the current model [36]. In contrast to bagging methods such as Random Forests [37], boosting focuses subsequent learners on remaining mistakes, which is effective for tabular feature sets with nonlinear interactions.

CatBoost employs ordered boosting to limit target leakage and reduce overfitting and uses symmetric (oblivious) trees that provide stable splits and fast inference [20,21]. Although CatBoost is often chosen for mixed numeric–categorical inputs, our features are entirely numeric (window-level summary and complexity metrics). This method remains suitable because it handles tabular numeric features with minimal preprocessing and performs reliably with default settings, as shown in [38,39,40].

Each sample is a fixed-length sliding window extracted from a simulated or empirical time series. The feature vector contains only numeric descriptors computed on that window. Importantly, the raw window values themselves are not used directly; instead, each window is first scaled to the unit interval before feature extraction, ensuring that the models operate on shape and temporal structure rather than absolute amplitude. This normalization is applied consistently across all window sizes and datasets, so the learned representations reflect relative dynamics within each window rather than global scale differences. We train two independent multi-class models on the same feature representation: one predicts the stability index α and one predicts the mean-reversion rate θ. The parameter grids for α and θ follow the simulation design defined earlier (Section 5). Training and test sets use the same grids but independent noise seeds, so test scores measure generalization to new noise realizations with the same statistics. Base series have length 2000 and are segmented into overlapping windows of length w∈{50, 100, 200, 250, 365} with shift w/2.

The models are implemented in the CatBoostClassifier library and are used with the library’s implementation of the multi-class loss and otherwise default hyperparameters, [20,21]. For each window length *w*, we train a separate pair of models (one for α and one for θ).

For each *w* and each target (α or θ), we construct a stratified split into 80% train and 20% test (stratification by the class label). From the training portion, we carve out an internal validation subset (10–20% of the training pool, stratified) used exclusively for early stopping and model selection.

We then perform a compact Bayesian hyperparameter search around the CatBoost GPU defaults using 3-fold cross-validation on the training set only. The search space centers on depth∈[5, 7], iterations∈[900, 1100], l2_leaf_reg∈[2, 5], and border_count∈[120, 136]; the objective is cross-validated accuracy. This yields a tuned candidate model. Between the baseline and the tuned candidate, we select the final model using performance on the internal validation data (or equivalently by cross-validated performance within the training set). The held-out test split is used only once, for the final evaluation. The chosen model is saved per window length and target.

Evaluation is performed once, on the held-out test split. We report per-class precision, recall, and $F_{1}$-score, overall accuracy, and weighted $F_{1}$-score. We also produce confusion matrices in two forms: absolute counts and row-normalized (to show conditional accuracy by true class). For the coarse screening use case, we reuse the same trained models without retraining to form binary tasks (Gaussian vs. non-Gaussian for α; mean reversion vs. no mean reversion for θ). Reproducibility is ensured by fixed parameter grids defined in the simulation section, documented seeds for training and testing generation, identical windowing and feature extraction across runs, and a consistent train/validation/test protocol per window size and target.

## 5. Initial Model Validation

We estimate two window-level quantities: the stability index α (tail heaviness) and the mean-reversion rate θ. Because both are local and depend on time scale, we evaluate models on sliding windows of fixed length w∈{50, 100, 250, 365}. Larger *w* provide more information per decision; smaller *w* provide finer time localization. Windows come from the dimensionless OU series generated on the α and θ grid defined in the simulation section (Section 3.2). Training and test sets use the same parameter grids but independent noise seeds, so test scores reflect generalization to new realizations with the same statistics.

**Figure 4:**
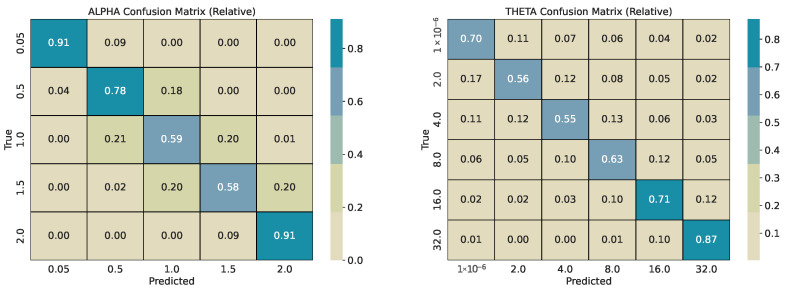
Row-normalized confusion matrices for the classification of α (**left**) and θ (**right**) using a window size of 50. Each row is normalized to sum to 1, so entries represent per-class conditional frequencies (i.e., the distribution of predicted labels given the true label).

**Figure 5:**
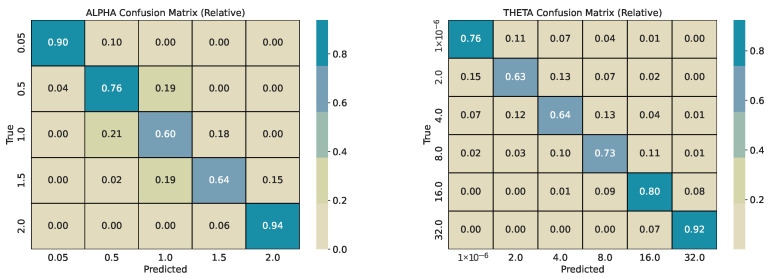
Row-normalized confusion matrices for the classification of α (**left**) and θ (**right**) using a window size of 100. Each row is normalized to sum to 1, so entries represent per-class conditional frequencies (i.e., the distribution of predicted labels given the true label).

**Figure 6:**
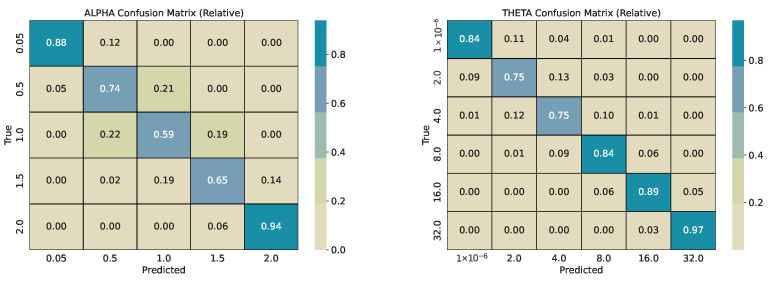
Row-normalized confusion matrices for the classification of α (**left**) and θ (**right**) using a window size of 250. Each row is normalized to sum to 1, so entries represent per-class conditional frequencies (i.e., the distribution of predicted labels given the true label).

**Figure 7:**
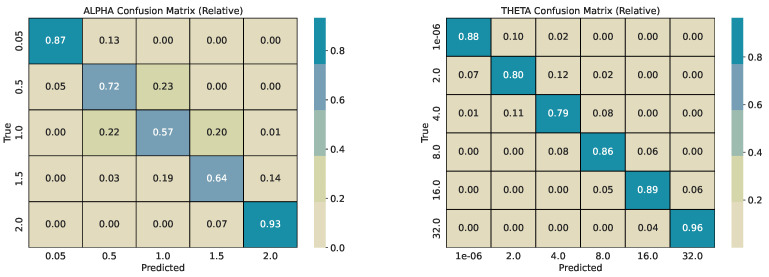
Row-normalized confusion matrices for the classification of α (**left**) and θ (**right**) using a window size of 365. Each row is normalized to sum to 1, so entries represent per-class conditional frequencies (i.e., the distribution of predicted labels given the true label).

Each base series (length 2000) is split into overlapping windows with shift w/2. Every window inherits the pair α and θ used for its source series. We train two CatBoost classifiers, one for α and one for θ, and evaluate them on a held-out test pool generated with different seeds. For every *w*, we report per-class precision, recall, and $F_{1}$-score, plus overall accuracy and weighted $F_{1}$-score. Classification reports are provided in Table 1, Table 2, Table 3 and Table 4. To describe error structure, we use row-normalized confusion matrices (Figure 4, Figure 5, Figure 6 and Figure 7).

Across window sizes, the α models reach mid-70% accuracy at *w* = 50 and remain close to that level at larger *w* in the five-class setup. The extremes α = 0.05 and α = 2.0 are classified best; intermediate α values show the most confusion with their neighbors. The θ models improve more with longer windows: accuracy rises from about 67% at *w* = 50 to about 84% at *w* = 250 and to about 86% at *w* = 365. The strongest reversion class θ = 32 has the highest per-class scores. Confusion is mostly between adjacent θ bins (e.g., 4 vs. 8 and 8 vs. 16), indicating ordinal errors, i.e., confusing processes similar in mean reversion, rather than arbitrary swaps.

Most misclassifications are concentrated in non-extreme regimes, where stochastic realizations generated by neighboring α and θ values exhibit strongly overlapping short-window statistics. In particular, intermediate tail indices (α∈{0.5, 1.0, 1.5}) and moderate mean-reversion rates (θ∈{2, 4, 8, 16}) can produce window-level trajectories that are visually and statistically indistinguishable at finite *w*, even under exact simulation. These confusions therefore reflect intrinsic identifiability limits of short stochastic segments rather than model instability, and they systematically diminish with increasing window length as more realizations of jumps or pull-to-mean behavior are observed. While this effect is clearly visible for θ, where both accuracy and diagonal concentration improve with increasing window size, no comparable reduction in confusion is observed for α. This is likely driven by a combination of decreasing effective training sample sizes at larger windows (due to fewer overlapping segments per base series) and the fact that intermediate α regimes remain statistically similar even at longer horizons. The persistence of roughly 75% accuracy across window sizes therefore suggests an intrinsic resolution limit for tail-index discrimination in finite windows, rather than insufficient model capacity.

The row-normalized confusion matrices show diagonal dominance at all window sizes and a tighter diagonal for θ as *w* grows. For α, longer windows increase the chance that rare, large deviations appear inside the window, which should improve the separation of very heavy-tailed and Gaussian regimes; however, the performance on identifying α correctly does not increase with increasing *w*. For θ, longer windows expose more of the pull-to-mean pattern or the lack of it, which improves the separation of reversion rates. Figure 4 illustrates the shortest-window setting, while Figure 6 and Figure 7 show the strongest separation for θ.

We also run a binary evaluation without retraining, using the same models and windows. For α, we group non-Gaussian states into a single “Lévy” class and test “Gaussian vs. Lévy”; for θ, we test “no mean reversion ($\theta =10^{-6}$) vs. mean reversion (all other θ).” Full binary reports are provided in Appendix A. In short, the Gaussian vs. Lévy task reaches about 94–96% accuracy even at *w* = 50, and the no-mean-reversion vs. mean-reversion task rises from about 89% to about 95% with window size. This indicates that the same trained models can be reused for coarse screening without additional fitting.

Overall, with independent-seed test sets and matched parameter grids, the models generalize consistently across window sizes. Multi-class errors are concentrated in neighboring labels, and the binary appendix confirms that the models provide reliable screening for Gaussian vs. non-Gaussian behavior and for mean reversion vs. no mean reversion.

**Summary.** The validation shows three main outcomes. First, the multi-class α classifier separates very heavy-tailed and Gaussian regimes reliably, with most errors confined to neighboring intermediate tail classes. Second, the multi-class θ classifier benefits strongly from longer windows, indicating that mean-reversion strength requires more temporal context than tail classification in this setup. Third, the same models achieve high accuracy on the two binary tasks, supporting their use as window-based detectors for Gaussian vs. non-Gaussian behavior and mean reversion vs. no mean reversion in analyses. For a window size of 365, the multi-class test performance is ${\mathrm{Acc}}_{\alpha }=0.7459$ ($F1_{\alpha }^{w}=0.7456$) and ${\mathrm{Acc}}_{\theta }=0.8642$ ($F1_{\theta }^{w}=0.8642$), consistent with stronger gains for θ at larger windows.

## 6. Complexity–Parameter Correlation Analysis for OU Windows

Another goal of this work is to quantify how well established time-series statistics (“complexity metrics”) reflect the generative parameters of the stochastic processes underlying our windowed data. We consider windowed segments generated from Ornstein–Uhlenbeck (OU)-type mean-reverting dynamics, as described in Section 3. In our experimental pipeline, OU-style trajectories are used as a controlled setting where two classes of properties are of primary interest:

- **Mean reversion/persistence** (captured by θ): statistics related to serial dependence, stationarity, and long-range structure (e.g., Hurst-type scaling or DFA-type exponents).
- **Tail heaviness** (captured by α): statistics from extreme value theory (EVT) and heavy-tail analysis (e.g., Hill-type estimators), as well as related distributional shape measures (e.g., kurtosis).

While the OU framework provides an interpretable baseline, the practical question is empirical: *which metrics correlate best with the known ground-truth parameters* α and θ across all datasets, and how does this compare to our supervised CatBoost predictors (described in the two previous sections) trained on the same windows?

To answer this, we evaluate a pool of 27 established time-series statistics and complexity metrics and compare their outputs against the known ground-truth parameters. This analysis is carried out separately for each window size *W* (i.e., W∈{50,100,250,365}) and for three representations of each window: (i) *scaled* windows (normalized windows used for ML), (ii) *unscaled* windows (original level series), and (iii) *return/increment* windows (first differences of the unscaled series).

### 6.1. Correlation and Linear Association Measures

This subsection describes how outputs from classical complexity metrics and from the CatBoost predictors are compared to known ground-truth parameters in the synthetic setting. The analysis is performed separately for the mean-reversion rate θ and the stability index α, and independently for each window size *W* and data representation (scaled levels, unscaled levels, and returns).

Let $Y=\{y^{\left(k\right)}\}$ denote the discrete set of ground-truth parameter values used in the simulations (either θ or α). For a fixed window size *W*, all windows generated from base series with ground truth $y^{\left(k\right)}$ are processed by a given estimator (classical metric or CatBoost predictor), yielding a collection of window-level outputs $\left\{{\hat{y}}_{i}\right\}$ associated with that ground-truth value.

Rather than evaluating correlations at the individual-window level, we aggregate estimator outputs by ground-truth class. Concretely, for each estimator, window size *W*, and ground-truth value $y^{\left(k\right)}$, we compute the sample mean

$${\bar{\hat{y}}}^{\left(k\right)}=\frac{1}{n_{k}}\sum_{i=1}^{n_{k}}{\hat{y}}_{i}$$

and the corresponding sample standard deviation, where $n_{k}$ denotes the number of windows available for that parameter value at the given window size. The correlation analysis is then performed between the vectors $\left\{y^{\left(k\right)}\right\}$ and $\left\{{\bar{\hat{y}}}^{\left(k\right)}\right\}$ across the discrete parameter grid. Error bars shown in correlation plots correspond to the empirical standard deviation within each ground-truth class and therefore visualize finite-window variability rather than estimation uncertainty in a parametric sense. We quantify association using three complementary measures: rank-based correlation, linear correlation, and an explicit linear recalibration fit.

**Spearman rank correlation.** Spearman’s ρ measures monotone association by applying Pearson correlation to rank-transformed samples. Let $\mathrm{rg}(y^{\left(k\right)})$ and $\mathrm{rg}({\bar{\hat{y}}}^{\left(k\right)})$ denote the ranks of the ground-truth values and the corresponding averaged estimator outputs. Then

(27) $$\rho_{S}=\mathrm{corr}\;\left(\mathrm{rg}\left(y\right),\mathrm{rg}\left(\bar{\hat{y}}\right)\right).$$

This measure is insensitive to nonlinear but monotone transformations and is therefore well suited for comparing proxy metrics to ground truth when only ordinal information is preserved.

**Pearson correlation.** Pearson’s *r* measures linear association between ground truth and averaged estimator outputs:

(28) $$r=\frac{\mathrm{cov}(y,\bar{\hat{y}})}{\sigma_{y}\sigma_{\bar{\hat{y}}}}.$$

Here, covariance and standard deviations are computed across the discrete ground-truth grid. Pearson correlation reflects both ordering and linear calibration quality.

**Linear recalibration and coefficient of determination.** For interpretability, we also fit a least-squares linear mapping between averaged estimator outputs and ground truth,

(29) $$y\approx a+b\;\bar{\hat{y}},$$

with closed-form coefficients

(30) $$b=\frac{\mathrm{cov}(\bar{\hat{y}},y)}{\mathrm{var}(\bar{\hat{y}})},\;a=\bar{y}-b\;\bar{\hat{y}}.$$

The goodness-of-fit is summarized by the coefficient of determination

(31) $$R^{2}=1-\frac{\sum_{k}{\left(y^{\left(k\right)}-\left(a+b\;{\bar{\hat{y}}}^{\left(k\right)}\right)\right)}^{2}}{\sum_{k}{\left(y^{\left(k\right)}-\bar{y}\right)}^{2}}.$$

This $R^{2}$ quantifies how much of the variance across the ground-truth grid is explained by a linear transformation of the estimator output.

Even though all three measures quantify association between ground truth and estimator outputs, they serve different and complementary purposes in this study. Spearman’s ρ evaluates whether the ordering of parameter values is preserved. This is important because many classical metrics act as qualitative proxies for mean reversion or tail heaviness: they may increase or decrease consistently with the true parameter without being linearly calibrated. In such cases, ordinal information is more relevant than exact proportional scaling.

Pearson’s *r* assesses linear association and therefore reflects both ordering and approximate calibration. In our framework, the estimators (classical metrics and CatBoost predictors) produce numerical outputs that are interpreted on a continuous scale. Evaluating linear correlation is therefore appropriate when the goal is to understand how well these outputs track the magnitude of the true parameter across the discrete grid.

Finally, the explicit linear recalibration provides a regression-based perspective. Since the synthetic ground truth is known and deterministic, we can examine how well a linear transformation of the estimator output reconstructs the parameter values. This is relevant because the workflow combines aspects of regression (predicting numerical parameters) with aspects of ordinal classification (distinguishing neighboring regimes).

In practice, stochastic processes with nearby parameter values can exhibit similar behavior, and different metrics may preserve ordering while differing in scale. Using Spearman, Pearson, and linear recalibration together therefore allows us to separate monotonic agreement, linear proportionality, and predictive calibration within one evaluation framework.

**Estimator-specific treatment.** For θ, classical metrics that produce persistence- or reversion-related quantities are mapped to $\hat{\theta }$ values via linear recalibration using Equations (29) and (30). For α, metrics are treated either as direct tail-index-like estimates (when applicable) or as monotone heavy-tail proxies.

The CatBoost predictors are evaluated using the same aggregation and correlation procedure. This places the machine learning outputs and classical estimators on equal footing and allows direct comparison of (i) ordinal consistency, (ii) linear calibration, and (iii) variability across windows within the same ground-truth regime.

Finally, because scaling and differencing alter fundamental signal properties, all correlations are computed separately for scaled windows, unscaled windows, and returns. This separation allows us to assess whether a given estimator primarily exploits amplitude information, shape information preserved under scaling, or increment-level structure.

The next subsection introduces the full set of metrics used, grouped by estimator family, and provides concise definitions and references for each.

### 6.2. Complexity and Statistical Metrics Used for Model Comparison

To complement the direct estimation of the OU parameters α and θ, we evaluate a pool of established time-series statistics and complexity measures that capture heavy-tail behavior, distributional shape, long-range dependence, mean-reversion proxies, entropy-based complexity, phase-space geometry, and spectral or low-rank structures. The goal is not to re-define these measures but to use them as *diagnostic descriptors* that can be compared against ground-truth α and θ labels and against the corresponding CatBoost predictions.

One metric is evaluated in more than one realization, i.e., different Hurst/scaling exponents. This is intentional, as different realizations can behave differently for short windows, scaled data, or increment/return series.

Table 5 summarizes all metrics used in the analysis, grouped by their primary interpretation and accompanied by a canonical reference. A detailed description of each metric is deferred to Appendix B.

### 6.3. Results: Correlation Analysis for W=100

**Figure 8:**
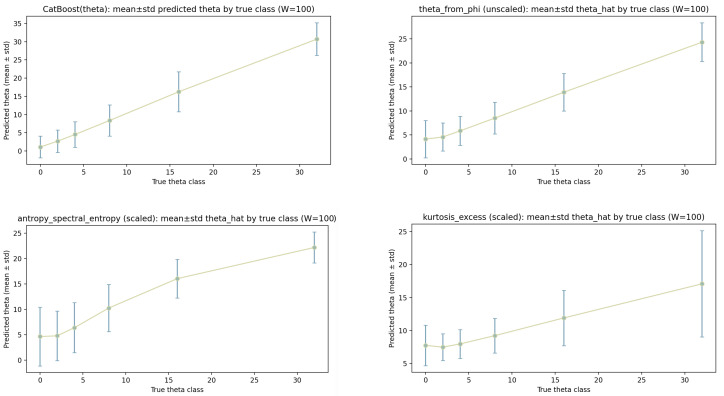
Calibration and correlation plots for the four strongest predictors of the OU mean-reversion parameter θ at window length W=100, evaluated on scaled-level data. The upper-left panel shows the CatBoost model predictions versus ground truth, achieving Spearman $\rho_{S}=0.973$, Pearson r=0.975, and linear $R^{2}=0.951$. The remaining panels show the best-performing classical metrics: the OU-consistent proxy theta_from_phi ($\rho_{S}=0.964$, r=0.985, $R^{2}=0.970$), spectral entropy ($\rho_{S}=0.738$, r=0.706, $R^{2}=0.499$), and excess kurtosis ($\rho_{S}=0.650$, r=0.654, $R^{2}=0.427$). All plots depict linear recalibration fits and illustrate the relative strength and structural differences between direct machine learning predictions and interpretable statistical surrogates.

**Figure 9:**
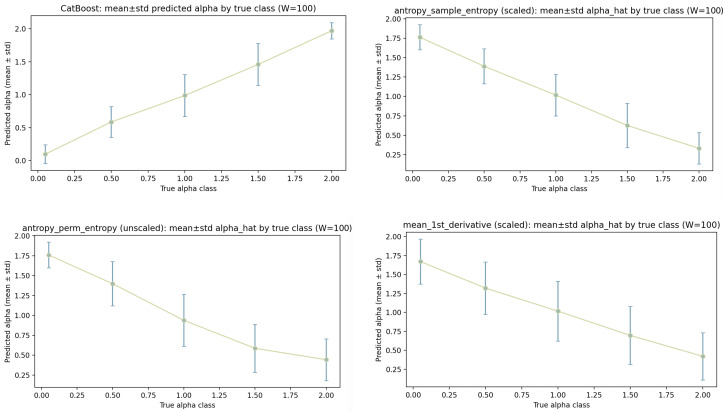
Calibration and correlation plots for the four strongest predictors of the parameter α at window length W=100, evaluated on scaled-level data. The upper-left panel shows the CatBoost model predictions versus ground truth, achieving Spearman $\rho_{S}=0.938$, Pearson r=0.937, and linear $R^{2}=0.878$. The remaining panels show the best-performing classical metrics (scaled): antropy_sample_entropy ($\rho_{S}=-0.910$, r=−0.909, $R^{2}=0.827$), antropy_perm_entropy ($\rho_{S}=-0.841$, r=−0.838, $R^{2}=0.703$), and mean_1st_derivative ($\rho_{S}=-0.785$, r=−0.784, $R^{2}=0.615$). All plots include linear recalibration fits and highlight the contrast between direct machine learning predictions and interpretable surrogate metrics, including the strong monotonic inverse associations observed for the entropy- and derivative-based features.

This section reports correlations between the OU parameters and a set of classical window statistics and contrasts them with CatBoost predictions. For each window, we compute each metric on three data variants: (i) min–max scaled levels, (ii) unscaled levels, and (iii) returns (first differences of the unscaled levels). For each parameter, the full correlation summaries are given in Table 6 (for θ) and Table 7 (for α). In addition, we provide four-panel calibration/correlation plots for each parameter (Figure 8 and Figure 9), showing CatBoost and the three strongest classical metrics selected from the scaled-level column of the corresponding table.

All calibration/correlation plots display the empirical relationship between a predictor (CatBoost output or a metric) and the corresponding ground-truth label. For each discrete ground-truth class (or bin), we compute the mean prediction and its standard deviation over all samples assigned to that class. The plotted points therefore represent class-conditional averages, and the error bars summarize within-class spread.

θ **(mean-reversion rate).** Table 6 shows that θ is best captured by metrics that are directly linked to OU mean reversion and by descriptors that reflect the regularity of the trajectory on the window. Among classical metrics on scaled levels, theta_from_phi provides the strongest and most consistent association with θ ($\rho_{S}=0.842$, r=0.895, $R^{2}=0.800$). This is expected because theta_from_phi is constructed from an AR(1) estimate ϕ and is therefore aligned with the discrete-time OU/AR(1) relation. The same metric retains essentially identical correlations on unscaled levels, indicating that the underlying estimator is largely invariant to affine rescaling of the levels in this setup. On returns, however, theta_from_phi degrades strongly ($\rho_{S}=0.027$, r=0.134, $R^{2}=0.018$), which is consistent with the fact that differencing removes the level-based mean-reversion structure and shifts the signal into a form that is not matched to an AR(1)-style estimation of mean reversion.

The second strongest scaled-level surrogate in Table 6 is spectral entropy, antropy_spectral_entropy ($\rho_{S}=0.773$, r=0.802, $R^{2}=0.644$). This suggests that the frequency-domain structure of the window is informative for θ: faster mean reversion tends to produce a trajectory with different spectral content than slow mean reversion, and this is reflected in an approximately monotonic, moderately linear relationship. In contrast to theta_from_phi, spectral entropy remains reasonably strong on unscaled levels ($\rho_{S}=0.807$, r=0.792, $R^{2}=0.628$) but is much weaker on returns ($\rho_{S}=0.155$, r=0.268, $R^{2}=0.072$), again indicating that differencing changes the features that relate to mean reversion.

Excess kurtosis, kurtosis_excess, provides a moderate but consistent relationship with θ on level data ($\rho_{S}=0.659$, r=0.623, $R^{2}=0.389$ for scaled; similar for unscaled). This indicates that the marginal distribution of the window values carries some information about the mean-reversion regime, but the relationship is weaker than for OU-consistent proxies and spectral structures. On returns, kurtosis_excess becomes negligible ($\rho_{S}=0.040$, r=0.006, $R^{2}\approx 0$), consistent with the fact that differencing changes the marginal distribution and reduces the direct link between the window-level distribution and the mean-reversion parameter.

Other metric families show limited suitability for θ at W=100. Heavy-tail estimators and tail indices (e.g., hill_index, xi_moment, xi_pickands) can exhibit moderate Spearman correlation on levels, but their Pearson correlation and $R^{2}$ are small to moderate (e.g., hill_index has $\rho_{S}\approx 0.666$ but r≈0.010 and $R^{2}\approx 0$), indicating nonlinear, weakly calibrated relationships that are not useful as quantitative surrogates for θ. Autocorrelation-based metrics as implemented here (e.g., lag1_autocorr, ar1_phi) show near-zero Pearson and $R^{2}$ in the table despite high Spearman values in some configurations; this pattern indicates that the rank ordering may be dominated by estimator artifacts or a restricted dynamic range, while the linear relationship to the label is absent.

The corresponding plot set in Figure 8 provides a qualitative view that matches the table. The CatBoost panel (upper-left) shows a tight, near-linear association with small within-class spread, consistent with strong $\rho_{S}$ and $R^{2}$ (Table row “CatBoost (model)”). For theta_from_phi, the class-mean points lie close to the recalibration line across the label range, and the error bars are comparatively small, indicating both good ranking and good calibration. For spectral entropy, the trend is monotonic but typically less linear than for theta_from_phi; the error bars are larger in parts of the range, indicating higher within-class variability and lower $R^{2}$. For excess kurtosis, the monotonic pattern is weaker and the scatter around the fit is larger, consistent with the moderate correlations and lower explanatory power.

α **(noise scale/volatility).** Table 7 shows a different structure for α than for θ. For α, the strongest classical predictors are entropy measures and simple derivative-based variability descriptors, while heavy-tail estimators provide only moderate correlations and are not consistently strong across preprocessing variants.

On scaled levels, the three strongest classical metrics are antropy_sample_entropy ($\rho_{S}=-0.910$, r=−0.909, $R^{2}=0.827$), antropy_perm_entropy ($\rho_{S}=-0.841$, r=−0.838, $R^{2}=0.703$), and mean_1st_derivative ($\rho_{S}=-0.785$, r=−0.784, $R^{2}=0.615$). The negative signs indicate that a larger α corresponds to lower entropy values under these definitions in the present setup. Across data variants, antropy_sample_entropy remains strong for unscaled levels and becomes even stronger on returns ($\rho_{S}=-0.937$, r=−0.937, $R^{2}=0.878$), suggesting that the noise scale is strongly reflected in the short-term irregularity of the increments. Similarly, antropy_perm_entropy remains strong across scaled and unscaled levels and stays substantial on returns, though weaker than sample entropy. In contrast, mean_1st_derivative is strong only on scaled levels and becomes weak on unscaled levels and returns, indicating sensitivity to scale and to how the derivative statistic is aggregated.

Classical “tail” descriptors show weaker and less stable performance for α. While kurtosis_excess increases in strength from scaled levels ($R^{2}=0.114$) to returns ($R^{2}=0.285$), it remains clearly below the entropy metrics. Tail-index estimators (e.g., hill_index, xi_pickands, xi_moment) reach moderate correlations in some variants (e.g., returns), but they do not approach the entropy-based surrogates. A practical interpretation is that OU trajectories with varying α primarily differ in short-scale irregularity and local variability rather than in stable heavy-tail structures within windows. Even when heavy-tail estimators correlate with α, they are not well suited for describing OU windows in a way that is robust and quantitatively calibrated across preprocessing, because OU increments are Gaussian in the idealized model and apparent tail effects can be driven by finite-sample effects, discretization, or conditioning on other parameters.

The four-panel plot in Figure 9 is consistent with this ranking. The CatBoost panel shows a strong, near-linear relationship with relatively small within-class standard deviations, consistent with the CatBoost row in Table 7 ($\rho_{S}=0.938$, r=0.937, $R^{2}=0.878$). The entropy panels show clear monotonic trends with points closely following the recalibration line across the label range, consistent with high |r| and large $R^{2}$. The error bars can widen for extreme classes, reflecting fewer windows per class and increased within-class variability, but the ordering remains stable. For mean_1st_derivative, the trend is still monotonic but shows more spread than the entropy metrics; the larger deviations from the fit line match the lower $R^{2}$ reported in the table.

**Summary and implications at W=100.** Across both parameters, the results separate metrics into (i) OU-consistent parameter proxies (most clearly for θ via theta_from_phi), (ii) regularity/complexity descriptors (entropy measures, most clearly for α), and (iii) distributional tail descriptors (heavy-tail and kurtosis measures). For θ, the strongest classical surrogate is the OU-consistent proxy, and most other metrics either lose signal on returns or show weak calibration. For α, entropy measures are the most stable and best calibrated surrogates across preprocessing variants, while heavy-tail estimators provide only moderate correlations and are not competitive with entropy-based descriptors in explaining the label variation. The calibration plots complement the correlation tables by showing where errors are concentrated (via within-class standard deviations) and whether deviations from linear recalibration are systematic (curvature) or predominantly noise (scatter around the fit).

**Cross-window robustness, i.e., W≠100.** The additional correlation tables in Appendix C confirm that the main conclusions at W=100 are not specific to a single window length. For α, Table A5, Table A6 and Table A7 show that entropy-based metrics remain the strongest and most stable classical surrogates across preprocessing variants, with consistently large-magnitude (typically negative) rank and linear correlations and high recalibration $R^{2}$ values; derivative-based metrics are often strong on scaled levels but are less stable across preprocessing, and heavy-tail estimators (e.g., Hill- and ξ-type estimators) can correlate with α—increasing with larger windows and on returns—but do not match the entropy features in calibration quality. For θ, Table A8, Table A9 and Table A10 show that the OU-consistent proxy theta_from_phi is the most reliable classical surrogate on level data and improves markedly with window length, while correlations on returns remain weak across window sizes, consistent with the loss of level-based mean-reversion information under differencing; secondary descriptors such as spectral entropy and excess kurtosis provide additional but weaker signal on levels. Across both parameters, CatBoost remains consistently strong and generally benefits from longer windows, while the calibration plots (mean prediction by ground-truth class with within-class standard deviation bars) provide a complementary view to the table metrics by indicating where dispersion is concentrated, whether the relation is approximately linear after recalibration, and whether the remaining deviations are mainly noise versus nonlinearity. Together, the results from Appendix C support the interpretation developed for W=100: α is primarily aligned with complexity/regularity descriptors (entropy and, to a lesser extent, derivatives), whereas θ is primarily aligned with OU-consistent persistence/mean-reversion structure captured by AR(1)-based proxies.

## 7. Daily Asset Data and Interval Comparison

Short-horizon distributional properties of financial returns are known to vary across market regimes and across assets. Heavy tails, volatility persistence, and state-dependent dependence structures are well-documented empirical features [2,60]. These characteristics can shift over time in response to changes in market microstructure, liquidity, or macroeconomic conditions [61]. Because our machine learning models infer tail behavior (α) and mean-reversion strength (θ) from short windows, applying them to long daily time series allows us to examine whether the relative prevalence of distributional regimes has changed across decades. This situates the method within the broader empirical literature on the evolving market structure and non-Gaussian return behavior.

We analyze daily end-of-day price series for four representative assets: Apple (AAPL), Microsoft (MSFT), the S&P 500 (GSPC), and the Dow Jones Industrial Average (DJI). The history is divided into two non-overlapping intervals:

Period 1:1995–2004,Period 2:2010–2024.

These intervals are selected to provide sufficiently long, contiguous samples for all assets and to contrast an earlier market environment with a more recent one. In practice, we chose them because they offer large and symmetric time spans with virtually no missing data for all four assets, i.e., primarily for reasons of data availability. All results are reported per asset and per interval.

**Figure 10:**
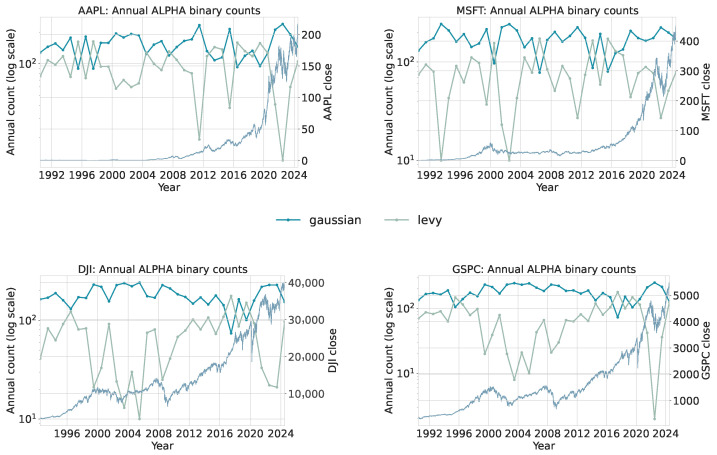
Annual counts of α regime labels (gaussian vs. levy) shown on a log scale.

**Figure 11:**
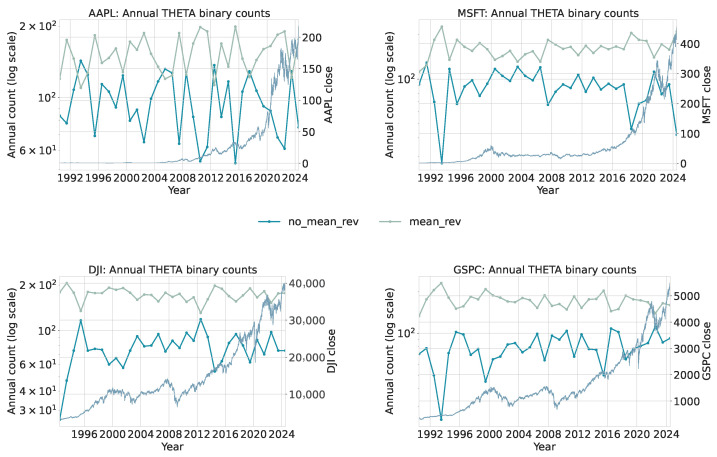
Annual counts of θ regime labels (mean reversion vs. no mean reversion) on a log scale.

To extract the local distributional structure, each series is scanned using a sliding window of length 50 with step size 1. For every window, the trained classifiers assign one category for the stability index α and one for the mean-reversion rate θ. This produces a categorical trace per asset. For each interval, we count how many windows fall into each discrete α or θ bin. Figure 10 and Figure 11 summarize the annualized distribution of category assignments, and Table 8 and Table 9 provide absolute bin counts for each asset and interval. The binary classification tables are collected in Appendix D.

Table 8 and Table 9 summarize the number of windows classified into each α or θ category. These counts provide a coarse view of how the empirical distribution of local regimes changes between intervals.

Interpretation.

Two consistent shifts appear when comparing Period 1 and Period 2. First, both broad indices (DJI and GSPC) show a notable reduction in windows classified as Gaussian (α=2.0), with a corresponding increase in α=1.5. AAPL and MSFT exhibit only modest changes. This indicates that index-level returns display heavier effective tails in the post-2010 sample, aligning with documented findings that aggregate markets can experience structural shifts in volatility and tail behavior [2,60].

Second, windows classified into the strongest mean-reversion state (θ=32) decline for all assets except MSFT. This shift is consistent with a regime in which large deviations occur more frequently and take longer to dissipate, reducing the prevalence of tight, high-frequency mean reversion. These patterns do not contradict informational efficiency: EMH does not require Gaussianity [62,63]. Rather, they indicate that distributional characteristics of returns can evolve even when markets remain informationally efficient.

## 8. Daily Sunspot Data and Interval Comparison

Short-term statistical properties of solar activity are known to vary across solar cycles, including changes in dispersion, intermittency, and persistence [22,64]. These variations reflect the underlying solar magnetic dynamo and its modulation across decades. Because our machine learning models quantify local tail behavior (α) and local mean-reversion strength (θ), applying the method to long daily sunspot records allows us to examine whether the prevalence of these regimes shifts systematically across different phases of solar activity. This links the analysis to empirical studies documenting structural differences between strong and weak solar cycles and motivates the use of short-window statistical diagnostics as complementary indicators of cycle-dependent behavior.

The daily international sunspot number series, published by SILSO (Sunspot Index and Long-term Solar Observations), provides a standardized measure of solar magnetic activity corrected for observational biases [65,66]. The time series reflects the emergence, evolution, and decay of sunspot groups and exhibits the well-known ∼11-year cycle. For this study, the record is divided into two non-overlapping intervals:

Period 1:1985–2004,Period 2:2005–2024.

These intervals were selected primarily based on data availability: they are long, contiguous segments with no missing observations and can be split into two approximately symmetric parts that permit a comparison between an earlier phase dominated by relatively strong solar cycles 22–23 and a later phase that includes the deep minimum between cycles 23 and 24, the comparatively weak solar cycle 24, and the early phase of solar cycle 25 [22,64,67]. All results are reported per interval. For clarity, the solar cycles discussed here span the following official SILSO/NOAA intervals: Cycle 22 (1986–1996), Cycle 23 (1996–2008), Cycle 24 (2008–2019), and Cycle 25 (2019–present).

**Figure 12:**
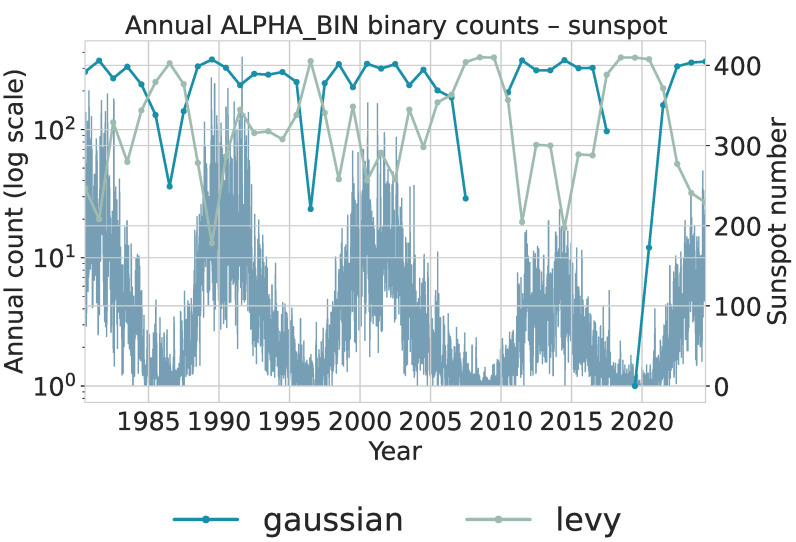
Annual binary counts for α regimes in the daily sunspot series. The plot shows α categories (gaussian vs. levy) on a logarithmic count scale, with the yearly average sunspot number overlaid for reference.

**Figure 13:**
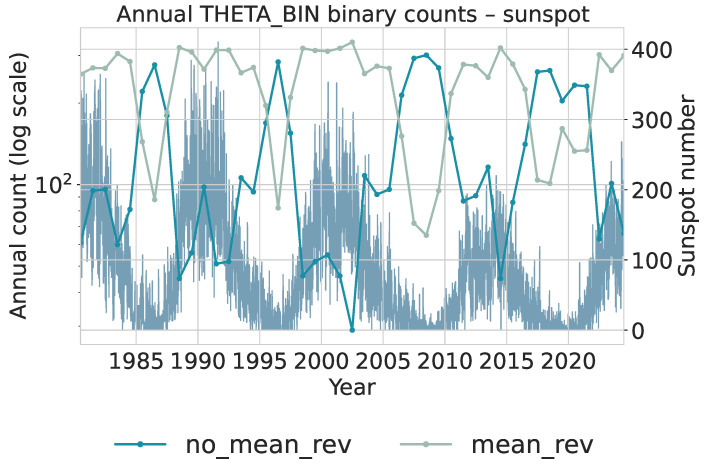
Annual binary counts for θ regimes in the daily sunspot series. The plot shows θ categories (mean_rev vs. no_mean_rev) on a logarithmic count scale, again with the yearly average sunspot number overlaid for reference.

To extract local statistical regimes, the daily series is scanned with a sliding window of length 50 and step size 1. Each window is assigned an α category (gaussian or levy) and a θ category (mean_rev or no_mean_rev) using the same trained models applied in the financial analysis. This produces two binary categorical sequences that summarize local tail behavior and local persistence. Annual counts of these binary categories are shown in Figure 12 for α and Figure 13 for θ. Both use logarithmic scaling for the category counts and overlay the yearly average sunspot number for reference. Figure 12: The gaussian category peaks near solar maxima, indicating that high-activity phases are dominated by many moderate, approximately symmetric fluctuations. In contrast, levy windows cluster more strongly around solar minima, where long quiet intervals are punctuated by intermittent larger changes, yielding heavier tails. The transition from cycles 22–23 to the weaker cycle 24 and early cycle 25 is visible as a relative increase in levy windows during the later decades, consistent with more irregular and intermittent activity.

Figure 13: Years with high sunspot activity are dominated by mean_rev windows, indicating that local deviations tend to be rapidly corrected during strong cycles. Around extended minima and in the weaker recent cycles, the share of no_mean_rev windows increases, reflecting a slower return toward equilibrium and more persistent deviations. The covariation in these counts with the sunspot cycle suggests that local mean-reversion strength is itself a cycle-dependent property of the solar dynamo.

Interval-level totals are reported in Table 10 and Table 11. These aggregated counts complement the annual dynamics by showing how the relative frequencies of distributional and dynamical regimes change across the two multi-cycle periods.

The earlier interval (1985–2004) is dominated by windows classified as Gaussian and mean reversion. This suggests that local increments tend to be moderate, symmetric, and quickly corrected, consistent with the relatively strong and regular solar cycles characterizing this period. High-activity cycles naturally generate many small fluctuations driven by frequent sunspot emergence, producing distributions closer to Gaussian and dynamics consistent with strong mean reversion.

The later interval (2005–2024) shows a clear increase in levy-classified windows, rising from 2500 to 3575. This shift indicates heavier-tailed short-term changes, consistent with the long quiet stretches and intermittent bursts observed in weaker and more irregular cycles (especially the deep minimum between cycles 23 and 24 and the comparatively weak cycle 24). The θ classification aligns with this pattern: the number of no_mean_rev windows increases substantially, implying that deviations persist longer and the process exhibits weaker corrective dynamics. Such behavior reflects the extended minima, asymmetric cycle shapes, and lower amplitudes documented in recent solar activity [22,64].

These findings form a coherent picture. Stronger cycles tend to generate frequent moderate fluctuations, leading to Gaussian-like and mean-reverting local dynamics. Weaker cycles produce intermittent, larger deviations that increase heavy-tailed behavior and decrease local mean reversion. Importantly, observing these differences is not only consistent with known solar cycle variability but also valuable for future inference. If only short-window statistics (α and θ) are available, the observed combination of heavier tails and weaker mean reversion provides evidence that the system is in a quieter and more irregular cycle, whereas predominantly Gaussian and mean-reverting behavior indicates a more active and regular phase. Since the intervals were intentionally chosen to contrast such regimes, the presence of clear statistical differences supports the usefulness of these local diagnostics for identifying the dynamical state of the solar cycle.

## 9. NASA POWER Daily Data for Austria

Daily reanalysis-based surface climate data provide a practical testbed for our regime-classification framework. They combine long, homogeneous time series with physically interpretable variables that are relevant for energy planning, agriculture, and impact studies [68,69,70]. Austria is a suitable case because it spans complex topography and continental-to-alpine climate gradients within a relatively small area, so that changes in short-horizon variability can be compared consistently across diverse conditions. By applying the α and θ classification to multi-decadal NASA POWER records, we assess whether the method recovers known contrasts between strongly forced and weather-modulated variables and whether it detects systematic shifts between two long, symmetric calendar intervals.

The Austrian surface-climate dataset analyzed here is derived from NASA’s POWER (Prediction Of Worldwide Energy Resources) project. POWER is an operational dissemination service hosted at NASA Langley Research Center that delivers solar and meteorological surface variables for energy, agrometeorology, and research applications, with consistent units, harmonized time standards, and long, gap-free coverage [68,70]. We retrieve daily records for a dense network of Austrian locations spanning all federal states—major urban centers (Vienna, Graz, Linz, Salzburg, Innsbruck, Klagenfurt, Bregenz), representative alpine valleys and high-elevation sites, and rural lowlands across Lower/Upper Austria, Styria, Carinthia, Tyrol, Vorarlberg, Salzburg, and Burgenland. Each site contributes a multi-decadal series from 1 January 1985 to 31 December 2024 for the variables used in this study: all-sky, clear-sky, and top-of-atmosphere shortwave irradiance; 10 m mean and daily maximum wind speed; 2 m relative humidity; bias-corrected daily precipitation total; surface pressure; diurnal temperature range; and 2 m dew/frost-point temperature. The Daily API and its query parameters are documented at https://power.larc.nasa.gov/docs/services/api/temporal/daily/ (accessed on 15 December 2025).

POWER provides point values by spatially sampling and interpolating authoritative gridded sources. For the parameters used here: (i) the *meteorological* variables (e.g., pressure, humidity, temperatures, winds, precipitation) originate from the NASA GMAO MERRA-2 reanalysis [69]; (ii) the *surface and TOA radiative* fields are sourced from the CERES (Clouds and the Earth’s Radiant Energy System) family of radiation products (CERES-SYN1deg and FLASHFlux), specialized for near-surface energy applications [71]. At delivery time, POWER harmonizes units—e.g., solar irradiance in MJ m^−2^day^−1^—and applies standard formatting and metadata. A concise provenance summary for POWER, which lists MERRA-2 for meteorology and CERES-SYN1deg/FLASHFlux for radiation, along with product lineages and links to upstream documentation, is provided at https://power.larc.nasa.gov/docs/methodology/data/sources/ (accessed on 15 December 2025).

Within POWER, PRECTOTCORR is a daily *bias-corrected* precipitation total designed for surface applications; radiation variables include ALLSKY_SFC_SW_DWN (all-sky surface shortwave on a horizontal plane), CLRSKY_SFC_SW_DWN (clear-sky surface shortwave), and TOA_SW_DWN (top-of-atmosphere shortwave), all provided as daily totals in MJ m^−2^day^−1^. Wind fields (WS10M, WS10M_MAX) are in m s^−1^, humidity in %, pressure in kPa, diurnal temperature range in °C, and dew/frost-point in °C. The POWER Parameter Dictionary documents the full variable set, naming, units, and community-specific conventions, and is the reference for parameter semantics used in this work: https://power.larc.nasa.gov/parameters/ (accessed on 15 December 2025).

From a data-generation standpoint, MERRA-2 is NASA’s modern global atmospheric reanalysis produced by the Global Modeling and Assimilation Office (GMAO). It assimilates a wide range of satellite and in situ observations into the GEOS modeling system to yield a spatially and temporally consistent estimate of the atmospheric state and fluxes from 1980 to the present [69]; POWER serves MERRA-2-based surface meteorology at a daily cadence suitable for agrometeorological and energy applications [68]. The POWER data-source documentation summarizes this linkage and provides upstream references to GMAO and the CERES radiation suite (SYN1deg for consistent climate records; FLASHFlux for low-latency fluxes). Users should consult that page for details on spatial resolution, updates, and cross-product consistency: https://power.larc.nasa.gov/docs/methodology/data/sources/ (accessed on 15 December 2025).

**Figure 14:**
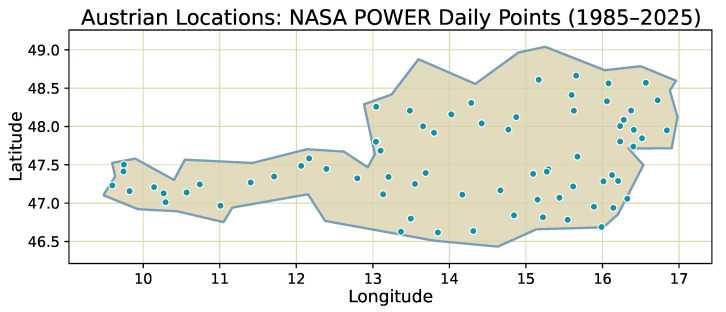
Spatial coverage of the Austrian NASA POWER network used in this study. Each marker represents one daily site record extending from 1985 to the end of 2024.

To characterize short-scale regime behavior consistently across variables and sites, each daily series is processed with a 50-day sliding window and step size of one day. For every window, we estimate the tail heaviness α and the mean reversion θ. Windows are assigned to discrete bins of these two quantities, and absolute counts per category are accumulated over two multi-decadal intervals, **1985–2004** and **2005–2025**. These intervals are chosen to be as long and symmetric as possible while maintaining uniform data availability for all locations and variables, so that changes in regime composition can be interpreted as temporal shifts rather than artifacts of coverage. The resulting distributions summarize how short-horizon dynamics change between the two periods, both for individual physical variables and for the full Austrian composite. A spatial overview of the analyzed locations is shown in Figure 14.

Following the spatial overview, we first present absolute counts by α and θ categories for all variables and both periods (Table 12 and Table 13). These tables provide the primary basis for comparing shifts in short-horizon regimes between the time periods 1985 to 2004 and 2005 to 2025. Complementary *binary* summaries that collapse categories into Gaussian vs. Lévy for α and mean-reverting vs. non-mean-reverting for θ are reported in the Appendix E (Table A13 and Table A14); they corroborate the main count patterns and are referenced in the discussion below.

**Figure 15:**
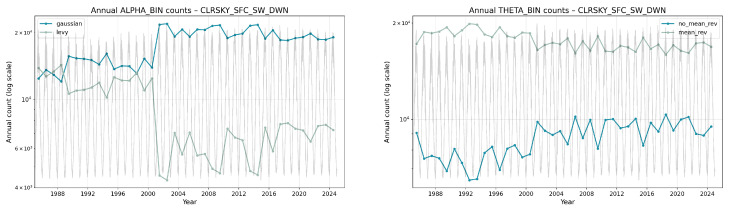
Annual window counts for CLRSKY_SFC_SW_DWN on a log scale. Left: α binary split (Gaussian vs. Lévy). Right: θ binary split (mean_rev vs. no_mean_rev). Both panels show a pronounced regime change around the year 2000, with a rising share of Gaussian and mean-reverting windows thereafter.

Figure 15 highlights a marked redistribution in CLRSKY_SFC_SW_DWN around the year 2000: the annual share of Gaussian windows increases while Lévy windows drop; in parallel, mean-reverting windows become more dominant relative to non-mean-reverting ones. This visual change is consistent with the pooled counts in Table 12 and Table 13 and with the binary summaries in the Appendix E (Table A13 and Table A14), where CLRSKY shows a higher α=2.0 mass and a relative tilt toward stronger reversion in the time span 2005 to 2025 compared to 1985 to 2004. The fact that these differences appear clearly in α and θ alone is useful for future applications: it implies that, when only short-window statistics are available, changes in the joint α and θ regime can be used as indicators of underlying shifts in the clear-sky radiative environment.

The aggregated counts across all Austrian locations reveal coherent and physically interpretable patterns. Radiative variables show strong contrasts between deterministic and weather-modulated components. The top-of-atmosphere shortwave flux (TOA_SW_DWN) concentrates almost entirely in the heaviest-tail side of the α grid and in the weakest-reversion bins of θ in both periods, reflecting the smooth but non-stationary seasonal cycle: windows drift slowly along the annual irradiance envelope with little tendency to revert to a local mean, producing very low θ values and an apparent departure from Gaussianity. In contrast, the surface-level shortwave components (ALLSKY_SFC_SW_DWN, CLRSKY_SFC_SW_DWN) allocate much larger mass to α=2.0 and to high θ, consistent with faster short-term equilibration driven by day-to-day weather variability. Between periods, small but systematic redistributions are visible: for instance, α=2.0 counts rise for CLRSKY (395,689 vs. 310,660) and θ=32 decreases slightly for ALLSKY (434,606 vs. 456,012), indicating a modest rebalancing between quickly mean-reverting, cloud-dominated variability and slowly varying seasonal components. These shifts are mirrored in Appendix E’s binary summaries, where CLRSKY shows more Gaussian and mean-reverting windows after 2005 (Table A13 and Table A14). Because these shifts are captured directly in α and θ, they demonstrate that the framework can distinguish changes in short-horizon radiative behavior between multi-decadal periods using only local window statistics.

Wind variables (WS10M, WS10M_MAX) remain dominated by α=2.0 and strong mean reversion in both intervals, consistent with near-Gaussian daily increments and rapid synoptic-scale adjustment typical of mid-latitude surface winds. The mild shift from α=2.0 toward α=1.5 after 2005 points to slightly heavier tails—occasional stronger gust events—but the θ profile remains heavily concentrated in the fastest reversion class, implying that such excursions decay quickly at the 50-day scale.

Precipitation (PRECTOTCORR) is distinct in showing persistent heavy tails with α concentrated around 1.0–1.5 and appreciable counts in 0.5. This reflects the intermittency and skewed nature of rainfall series, where dry spells alternate with bursts of wet days. Its θ profile, while still anchored by high-reversion bins, includes non-trivial counts at weaker θ, consistent with multi-day weather regimes or blocked patterns that maintain elevated precipitation probabilities over several days.

Thermodynamic fields—surface pressure, relative humidity, dew-point temperature, and diurnal temperature range—show the opposite signature. These variables concentrate almost entirely in the Gaussian, fast-reverting regime. Surface pressure and humidity display high θ and α=2.0, reflecting small, symmetric day-to-day fluctuations about a slowly varying mean. Dew-point temperature behaves similarly but with slightly broader tails, while the diurnal range exhibits the strongest mean reversion of all variables, with more than $5.1\times 10^{5}$ windows classified at θ=32 in each period, indicating extremely rapid recovery of short-term anomalies.

Across all variables, three consistent themes emerge. Deterministically forced quantities such as top-of-atmosphere radiation remain structurally heavy-tailed and weakly reverting at this window scale. Weather-modulated fields—radiation near the surface, wind, and precipitation—occupy intermediate regimes where the balance between intermittency and reversion depends on local processes and changes only subtly between the two periods. Thermodynamic quantities remain stably Gaussian and fast-reverting, with minimal redistribution across decades. These patterns conform to established statistical characteristics of daily meteorological data and show that NASA POWER, with harmonized MERRA-2 inputs, reproduces these relations consistently across space and time. In this sense, the α and θ representation functions as a compact tool for analyzing short-horizon variability and for inferring regime shifts from local window statistics alone.

## 10. Discussion

Our goal is to recover two local, window-scale descriptors of dynamics, stability α (tail heaviness) and the mean-reversion rate θ, and to show that the same trained classifiers are useful across synthetic benchmarks and three real-world domains. Methodologically, the approach is best viewed as a *tool*: given a univariate time series and a choice of window length, it produces a categorical description of tail behavior and recovery speed without re-estimating a full stochastic model for each segment. The synthetic experiments establish that this tool can separate classes on controlled data; the financial, solar, and climate applications then illustrate how the same instrument behaves in applied settings, where “ground truth” is partial and domain knowledge must guide interpretation.

On **synthetic, dimensionless Ornstein–Uhlenbeck processes** with α-stable increments, we train separate CatBoost models for α and θ on windows of length w∈{50,100,250,365}. The multi-class reports in Table 1, Table 2, Table 3 and Table 4 and the confusion matrices in Figure 4, Figure 5, Figure 6 and Figure 7 show consistent generalization: accuracies for θ increase with *w*, accuracy for α stays at ≈75%, errors are predominantly between adjacent classes, and extremes are easiest to identify (e.g., α=2 and α=0.05; $\theta \;=\;10^{-6}$ and θ=32). The auxiliary binary evaluation (Appendix A) confirms that the same models, without retraining, act as reliable screeners: “Gaussian vs. Lévy” for α reaches roughly 94–96% accuracy at w=50 and higher at longer windows, while “no mean reversion vs. mean reversion” for θ rises from about 89% (w=50) to about 97% at w=365). These outcomes provide a controlled baseline for interpreting the real-data applications.

Beyond classification accuracy, the complexity–parameter correlation analysis (Section 6) clarifies which window-level statistics align with the OU parameters and how this depends on preprocessing. At W=100, θ is best captured by OU-consistent, AR(1)-based surrogates on level data, with theta_from_phi providing the strongest and most linearly calibrated classical association (Table 6, Figure 8). Spectral entropy contributes secondary but meaningful signal on levels, indicating that mean reversion affects the spectral distribution of windowed trajectories, while most metrics—especially OU-consistent ones—degrade on returns, consistent with differencing, removing the level-based mean-reversion structure. For α, the ordering differs: entropy-based irregularity measures dominate, with sample and permutation entropy yielding the strongest and most stable associations across scaled levels, unscaled levels, and (for sample entropy) returns (Table 7, Figure 9). Across both parameters, the calibration plots show that residual errors are primarily reflected in within-class spread rather than strong systematic nonlinearity, supporting linear recalibration as an adequate summary for the leading metrics.

The appendix results across window sizes (Appendix C) confirm that these conclusions are robust to *W*. For θ, theta_from_phi remains the most reliable classical surrogate on level data and strengthens with larger windows, while correlations on returns remain weak. For α, entropy measures consistently outperform tail-index estimators across *W*, whereas EVT-style metrics and tail-shape proxies improve with larger windows and on returns but remain less well calibrated. Overall, this analysis provides an internal consistency check for the classifier-based results: CatBoost remains more accurate and better calibrated than any single classical metric, while the metric comparisons clarify the dominant information sources—level-based mean-reversion structure for θ and short-scale irregularity for α. This supports the interpretation used throughout the applications, where shifts in θ reflect changes in recovery dynamics and shifts in α reflect changes in the prevalence of irregular, burst-like behavior.

Applied to daily **financial series (AAPL, MSFT, DJI, GSPC; Section 7)**, the window-level α and θ maps indicate a post-2010 redistribution toward heavier effective tails and fewer occurrences of the strongest mean-reversion band in the broad indices, with more modest shifts for the two stocks. Concretely, α=2.0 counts fall for GSPC and DJI between 1995–2004 and 2010–2024, with mass moving largely to α=1.5 (Table 8); in parallel, θ=32 counts decline for the indices and for AAPL, while MSFT remains closer to flat (Table 9). The annual traces in Figure 10 and Figure 11 visualize the same pattern. This is compatible with established “stylized facts” of asset returns, such as fat tails, volatility clustering, and scale-dependent dependence [2,7,52], and it does not contradict informational efficiency [62,63]. Rather, it echoes classic variance-ratio evidence that equity returns are positively autocorrelated at short horizons but negatively autocorrelated at multi-year horizons, implying a sizable transitory component and mean reversion over longer windows [9,52,72]. In our taxonomy, more windows labeled as heavy-tailed and fewer labeled as very fast-reverting mean that outlier moves occur more often and take longer to dissipate, especially in the indices after 2010.

A distinct episode visible across all annual α and θ traces is the COVID-19 period (2020–2021). In each of the MSFT, GSPC, DJI, and AAPL panels, the onset of the pandemic coincides with a sharp increase in windows classified as heavy-tailed and a concurrent reduction in strongly mean-reverting windows, reflecting the abrupt and persistent volatility associated with the global shutdown and subsequent market reactions. The α-based counts show a temporary shift toward the Lévy side, while the θ-based counts display fewer fast-reversion labels, consistent with slower dissipation of shocks during crisis conditions. This pattern is observable in all four assets despite their different long-term trajectories, indicating that the classifiers capture a coherent, system-wide regime disturbance. The clear COVID-19 signal illustrates that the method can detect short-horizon structural breaks and extreme periods directly from window-level dynamics, reinforcing its suitability as a tool for identifying regime changes in high-frequency financial time series.

Here, the role of the tool is exploratory: it condenses large price archives into local “regime maps” that are consistent with known properties but are not tied to a single structural event.

For **daily sunspot numbers (SILSO; Section 8)**, the two-interval comparison (1985–2004 vs. 2005–2024) shows a clear shift away from Gaussian-like windows toward heavier tails and a concurrent weakening of mean reversion. Interval counts move from 4805 Gaussian vs. 2500 Lévy in the time span from 1985 to 2004 to 3730 vs. 3575 in the next period from 2005 to 2024 (Table 10), while no_mean_rev rises from 2216 to 3311 windows (Table 11). The annual binary plots in Figure 12 and Figure 13 show that Gaussian and mean-reverting windows cluster near cycle maxima, whereas Lévy and non-reverting windows increase near minima. Interpreting the pair α and θ together, higher-activity cycles yield many small, quickly corrected fluctuations (more Gaussian and more mean reversion), while the weaker and more irregular cycles after 2005 present longer durations interrupted by intermittent bursts (heavier tails and weaker reversion).

For the **Austrian NASA POWER dataset (Section 9),** the accumulated counts across locations and variables (Table 12 and Table 13) separate three clear classes of behavior. The top-of-atmosphere shortwave downward irradiance (TOA_SW_DWN), which represents the incoming solar radiation before any atmospheric interaction, is governed almost entirely by deterministic orbital geometry. As a consequence, its 50-day windows fall predominantly into heavy-tailed, weak-reversion bins: within such short windows, the signal follows the seasonal cycle and exhibits slow drift with little short-term corrective dynamics. Surface-level shortwave irradiance under all-sky conditions (ALLSKY_SFC_SW_DWN) and clear-sky conditions (CLRSKY_SFC_SW_DWN), together with near-surface wind speed (WS10M, WS10M_MAX) and daily precipitation totals (PRECTOTCORR), reflects weather-modulated processes. Surface irradiance and wind fields show largely Gaussian, fast-reverting windows, indicating frequent short-term adjustments driven by synoptic variability. In contrast, precipitation exhibits persistent heavy tails and non-negligible counts in weaker-reversion classes, consistent with the intermittent alternation between dry spells and wet episodes typical of mid-latitude rainfall. Thermodynamic variables—surface pressure (PS), relative humidity at 2 m (RH2M), dew-point temperature (T2MDEW)—and the diurnal temperature range (T2M_RANGE) exhibit the opposite pattern. These quantities remain almost exclusively in the Gaussian, strongly mean-reverting regime, reflecting tight physical constraints and rapid synoptic adjustment. Their distributions show little redistribution across decades, consistent with their stable dynamical control. A notable finding is the redistribution in clear-sky surface shortwave irradiance (CLRSKY_SFC_SW_DWN) around the year 2000, visible in the annual binary splits (Figure 15). After 2000, windows labeled as Gaussian and mean-reverting become more frequent. This shift aligns with the period counts and binary summaries (Table A13 and Table A14). While not attributable to a single documented forcing event, the pattern illustrates how the method distinguishes between (i) deterministic seasonal drift (TOA irradiance), (ii) weather-driven variability (surface irradiance, winds, precipitation), and (iii) strongly regulated thermodynamic fields, using one consistent window-level diagnostic framework.

Across these three use cases, the role of the examples is therefore complementary. Financial series test whether the method recovers and refines well-known stylized facts. Sunspots provide a case where independent physical knowledge of cycle changes allows a qualitative external check. The Austrian climate fields illustrate how the same classifiers behave on multi-variable, spatially distributed data and how they separate deterministic and stochastic components. In all three settings, the tool does not “prove” specific causal stories; rather, it produces compact, interpretable summaries that can be compared with external knowledge and used to formulate hypotheses about regime changes, resilience, and control.

Across all domains, classifying θ and α provides an operational summary: treat θ as a recovery meter and α as a tail-risk meter. Trends toward lower θ with stable or heavier tails warn of slower recovery and more persistent deviations; trends toward higher θ with lighter tails indicate stabilization. Mixed signals call for domain checks (e.g., coupling changes, controls, measurement). This framing is consistent with early-warning theory in complex systems: as systems approach critical transitions, recovery slows, autocorrelation rises, and variance inflates [73,74,75]. Our window-level θ labels operationalize recovery speed, while α labels track the potential for large shocks; together they translate information about a system’s resilience into measurable segments. Finally, our sensitivity analyses on synthetic data highlight an inherent limitation: finite windows impose unavoidable uncertainty, particularly for intermediate classes where local statistics overlap; increasing *w* reduces but does not eliminate near-neighbor confusions. In sum, the synthetic benchmarks show separability and accuracy of our approach, and the three real-world applications show that the same α and θ classifiers produce interpretable, domain-consistent classifications of tail behavior and recovery speed across markets, solar activity, and surface climate.

### Larger-Scope Interpretation: Linking α and θ to Real-World Systems

Our two parameters, the stability index α and the mean-reversion rate θ, summarize complementary dimensions of local dynamics: how large typical fluctuations are, and how quickly a system returns toward equilibrium after a disturbance. Reading them together allows us to link the window-level patterns found in financial, solar, and climatic time series to broader mechanisms of variability, resilience, and control across complex systems.

When θ is large, perturbations decay rapidly and the system quickly reverts to baseline; when θ is small, deviations persist, and recovery slows. In the literature on critical slowing down, such declines in recovery speed and increases in short-lag autocorrelation have been interpreted as early-warning indicators of approaching tipping points [73,74,75]. In our analysis, extended runs of persistent mean-reversion windows with declining effective θ therefore suggest weakening resilience, whereas stable or increasing θ implies a regime of fast equilibration. The results across all domains fit this intuition: synthetic and real systems with strong restoring forces, e.g., atmospheric pressure or diurnal temperature range, remain predominantly high-θ; those with slower or state-dependent feedbacks, e.g., precipitation, financial volatility, or solar activity, show more mass in weak-reversion bins and longer persistence of deviations.

The stability index α quantifies the thickness of the tails: values near 2 indicate Gaussian-like noise with rare extremes, while a smaller α marks increasingly heavy tails, with a higher probability of large jumps [2,7]. Heavy-tailed windows capture episodes where the variance of innovations is effectively infinite, implying bursts, jumps, or clustered extremes rather than steady diffusion. In markets, this corresponds to volatility clustering and drawdowns; in solar or climatic series, it maps to intermittent high-amplitude events (solar storms and intense precipitation). A shift toward higher α may signal stabilization or averaging (e.g., due to stronger controls or measurement smoothing), while a decline toward lower α points to more erratic, bursty regimes. We therefore interpret α only jointly with θ: persistent heavy tails with low θ imply systems that both generate and retain large deviations, whereas heavy tails with high θ indicate frequent shocks but rapid recovery.

The financial results follow this logic. Equity returns show the well-documented pattern of positive short-horizon autocorrelation and negative long-horizon autocorrelation, implying transitory components and mean reversion [9,52,62,63,72]. In our maps, such behavior appears as alternating patches of mean_rev and no_mean_rev windows. The increase in heavy-tailed, weakly reverting intervals in indices after 2010 (Section 7) corresponds to periods of higher volatility and slower dissipation of shocks—consistent with post-crisis market structure changes and the persistence of macro-volatility. Individual stocks, particularly AAPL and MSFT, show similar but less pronounced effects, suggesting partial insulation due to firm-specific growth factors.

The sunspot record provides a natural analog with a clear physical driver. Around each 11-year solar cycle maximum, the series exhibits many small, quickly corrected fluctuations, producing high α and high θ; near minima, activity becomes intermittent and clustered, lowering both parameters. Our counts and annual traces (Section 8) show that after 2005, the weaker, more irregular cycles coincide with a rise in Lévy and non-reverting windows, indicating a slower and more uneven recovery of solar activity after shocks. This matches known asymmetries and intermittency in modern solar cycles and supports interpreting θ as a dynamical recovery metric beyond classical variance measures.

The NASA POWER results (Section 9) extend this view to terrestrial climate fields. Deterministically forced quantities such as top-of-atmosphere shortwave flux concentrate in low-θ, heavy-tail bins, reflecting smooth seasonal drift with minimal stochastic correction. Near-surface variables—irradiance under clouds, winds, precipitation—occupy intermediate regimes where fast meteorological feedbacks coexist with intermittent forcing. The sharp redistribution in clear-sky surface shortwave irradiance (CLRSKY_SFC_SW_DWN) around 2000 (Figure 15) shows how large-scale radiative changes project onto local tail and reversion patterns: the post-2000 era displays more Gaussian and mean-reverting windows, potentially reflecting shifts in aerosol loading or cloud climatology. Thermodynamic fields such as pressure and humidity remain stably Gaussian and rapidly reverting, as expected under strong synoptic control. The binary appendices (Table A13 and Table A14) confirm that these categories are consistent across space and decades.

Taken together, the cross-domain synthesis suggests a simple operational reading. Treat θ as a *recovery meter* and α as a *tail-risk meter*. Moving toward lower θ with stable or heavier tails signals slower recovery and higher persistence of shocks—an early-warning pattern observed in many complex systems [73,74]. Moving toward higher θ with lighter tails denotes stabilization and stronger feedback. Mixed signals, such as heavier tails but faster recovery, call for domain-specific interpretation (e.g., improved buffering or new controls masking underlying volatility). This joint perspective avoids over-interpreting any single metric and links our window-level classifications to the macroscopic behavior of markets, solar cycles, and climate systems. It also clarifies the role of the method in practice: not as a stand-alone detector of specific events but as a compact diagnostic tool that turns long time series into interpretable maps of variability, recovery, and systemic risk.

At the same time, we need to emphasize here that the real-world applications do not provide a quantitative ground truth and are therefore not used as direct accuracy benchmarks. To address this limitation, we added a dedicated section (Section 6) that compares the classifier outputs to established classical estimators and complexity metrics in the synthetic setting, where our ground truth is known, thus connecting well-established metrics and our heavy-tail and mean-reversion proxies.

## 11. Conclusions

We introduced a method to estimate the local mean-reversion rate θ and the stability index α from time series using a feature-based, supervised learning framework trained on synthetic Ornstein–Uhlenbeck paths with α-stable increments. The approach combines interpretable statistical descriptors with gradient-boosted classifiers to assign each short window of a real series to discrete α and θ categories. Synthetic benchmarks confirm that both parameters can be recovered with high accuracy and that confusion is largely confined to adjacent classes across window lengths.

Applying the same models to financial, solar, and climatic datasets shows that α and θ capture different aspects of system dynamics in a consistent way. In financial returns, we find a redistribution between 1995–2004 and 2010–2024 toward heavier effective tails and fewer occurrences of the strongest mean-reversion band in the broad indices, with smaller changes for AAPL and MSFT. In solar activity, the comparison between 1985–2004 and 2005–2024 reveals that the weaker and more irregular recent cycles exhibit more Lévy-type and non-mean-reverting windows, while Gaussian and mean-reverting windows cluster around earlier cycle maxima. In the Austrian NASA POWER climate data, atmosphere radiation remains structurally heavy-tailed and weakly reverting, near-surface radiative and wind fields are largely Gaussian and fast-reverting, and thermodynamic variables are stably Gaussian and strongly mean-reverting; a clear redistribution in clear-sky shortwave irradiance around 2000 illustrates how large-scale radiative changes project onto α and θ, respectively. In all three use cases, the method does not replace detailed physical or econometric modeling but acts as a compact diagnostic that produces domain-consistent categorizations of tail behavior and recovery speed.

From a methodological perspective, the main contribution lies in demonstrating that discrete, window-level recovery and tail-risk regimes can be inferred reliably from short segments, without explicit parametric fitting on each window. This bridges a practical gap between classical asymptotic estimators and real-world applications where regime changes occur on time scales too short for stable traditional inference.

The comparison with established complexity and heavy-tail estimators clarifies the information content exploited by the classifiers: short-scale irregularity and entropy dominate practical α discrimination on finite windows, while classical extreme value theory tail-index estimators require substantially longer segments to become competitive. For θ, simple OU-consistent persistence measures already capture most of the relevant signal, and the machine learning models can be viewed as robust, window-adaptive aggregations of these classical ideas.

Conceptually, the two parameters provide a simple operational reading: θ acts as a recovery meter and α as a tail-risk meter. Trends toward lower θ with stable or heavier tails signal slower recovery and more persistent deviations, whereas higher θ with lighter tails indicates faster equilibration and reduced local tail risk. Because the classifiers operate directly on short windows and require only a generic feature representation, the framework is portable across domains and suitable for scanning long observational records for changes across short-horizon regimes.

This study has several limitations. First, the classifiers are trained on a specific synthetic family (dimensionless Ornstein–Uhlenbeck processes with symmetric α-stable noise), so applications to systems with qualitatively different dynamics or strong asymmetries should be interpreted as phenomenological mappings rather than structural parameter recovery. Second, we work with finite window lengths and a discrete grid of α and θ classes; windows near class boundaries are intrinsically hard to distinguish, and some loss of information relative to continuous parameter estimates is unavoidable. Third, the feature set and CatBoost models were chosen for robustness and practicality, not optimality; other feature constructions or architectures might capture additional structures or yield different decision boundaries. Finally, all real-data case studies rely on external data products (financial indices, SILSO sunspot numbers, NASA POWER fields) with their own uncertainties and revisions, so our labels reflect both underlying dynamics and data-processing choices. These constraints do not invalidate the results but should be kept in mind when transferring the method to new domains or when using α and θ labels for operational decisions.

Future work should extend this approach to multivariate and spatiotemporal settings, integrate uncertainty estimates for the α and θ assignments, and investigate whether systematic shifts in the joint distribution of these labels can serve as early indicators of emerging transitions in economic, heliophysical, or climate-related systems.

The full code to reproduce our findings together with the trained models is available at https://github.com/Raubkatz/HeavyTailsMeanReversion (accessed on 15 December 2025).

## Figures and Tables

**Table 1:** Classification reports for sliding window of **50 points**.

α Classification				
**Class**	**Prec.**	**Recall**	**F1-Score**	**Support**
0.05	0.96	0.91	0.94	948,000
0.5	0.71	0.78	0.74	948,000
1.0	0.60	0.59	0.59	948,000
1.5	0.66	0.58	0.62	948,000
2.0	0.81	0.91	0.86	948,000
**Accuracy**	0.7520			
**Weighted F1-Score**	0.7497			
**θ Classification**				
**Class**	**Prec.**	**Recall**	**F1-Score**	**Support**
$10^{-6}$	0.66	0.70	0.68	790,000
2.0	0.66	0.56	0.60	790,000
4.0	0.63	0.55	0.59	790,000
8.0	0.62	0.63	0.63	790,000
16.0	0.65	0.71	0.68	790,000
32.0	0.79	0.87	0.83	790,000
**Accuracy**	0.6706			
**Weighted F1-Score**	0.6674			

**Table 2:** Classification reports for sliding window of **100 points**.

α Classification				
**Class**	**Prec.**	**Recall**	**F1-Score**	**Support**
0.05	0.95	0.90	0.92	468,000
0.5	0.70	0.76	0.73	468,000
1.0	0.61	0.60	0.61	468,000
1.5	0.72	0.64	0.68	468,000
2.0	0.86	0.94	0.90	468,000
**Accuracy**	0.7681			
**Weighted F1-Score**	0.7670			
**θ Classification**				
**Class**	**Prec.**	**Recall**	**F1-Score**	**Support**
$10^{-6}$	0.76	0.76	0.76	390,000
2.0	0.70	0.63	0.66	390,000
4.0	0.67	0.64	0.65	390,000
8.0	0.68	0.73	0.70	390,000
16.0	0.76	0.80	0.78	390,000
32.0	0.90	0.92	0.91	390,000
**Accuracy**	0.7457			
**Weighted F1-Score**	0.7442			

**Table 3:** Classification reports for sliding window of **250 points**.

α Classification				
**Class**	**Prec.**	**Recall**	**F1-Score**	**Support**
0.05	0.95	0.88	0.91	180,000
0.5	0.67	0.74	0.70	180,000
1.0	0.59	0.59	0.59	180,000
1.5	0.72	0.65	0.68	180,000
2.0	0.87	0.94	0.90	180,000
**Accuracy**	0.7583			
**Weighted F1-Score**	0.7579			
**θ Classification**				
**Class**	**Prec.**	**Recall**	**F1-Score**	**Support**
$10^{-6}$	0.89	0.84	0.86	150,000
2.0	0.76	0.75	0.75	150,000
4.0	0.75	0.75	0.75	150,000
8.0	0.81	0.84	0.82	150,000
16.0	0.89	0.89	0.89	150,000
32.0	0.94	0.97	0.96	150,000
**Accuracy**	0.8392			
**Weighted F1-Score**	0.8391			

**Table 4:** Classification reports for sliding window of **365 points**.

α Classification				
**Class**	**Prec.**	**Recall**	**F1-Score**	**Support**
0.05	0.94	0.87	0.90	108,000
0.5	0.66	0.72	0.69	108,000
1.0	0.58	0.57	0.57	108,000
1.5	0.70	0.64	0.67	108,000
2.0	0.86	0.93	0.90	108,000
**Accuracy**	0.7459			
**Weighted F1-Score**	0.7456			
**θ Classification**				
**Class**	**Prec.**	**Recall**	**F1-Score**	**Support**
$10^{-6}$	0.92	0.88	0.90	90,000
2.0	0.79	0.80	0.79	90,000
4.0	0.79	0.79	0.79	90,000
8.0	0.85	0.86	0.86	90,000
16.0	0.90	0.89	0.90	90,000
32.0	0.94	0.96	0.95	90,000
**Accuracy**	0.8642			
**Weighted F1-Score**	0.8642			

**Table 5:** Statistical and complexity metrics used for correlation analysis, grouped by interpretation.

Metric (Library Name)	Interpretation/Keyword	Reference
**Tail behavior and extreme value theory**		
hill_index	Pareto tail-index estimator	[41]
xi_pickands	Extreme value index ξ	[42]
xi_moment	Moment-based EVI estimator	[43]
tail_rate	Tail exceedance frequency (quantile-based)	[44]
skewness	Distributional asymmetry	[45]
kurtosis_excess	Tail weight (fourth moment)	[45]
**Mean reversion, memory, and scaling**		
hurst_h	Hurst exponent *H*	[46,47]
hurst_catboost_ml	ML-based Hurst proxy	[39]
dfa_alpha	DFA scaling exponent	[48,49,50]
lag1_autocorr	Lag-1 autocorrelation	[51]
ar1_phi	AR(1) persistence parameter	[51]
theta_from_phi	OU mean-reversion rate (via AR(1) mapping)	[1]
half_life_from_phi	Mean-reversion half-life	[1]
variance_ratio_q2	Variance-ratio test (*q* = 2)	[52]
variance_ratio_q5	Variance-ratio test (*q* = 5)	[52]
adf_stat	Unit-root test statistic	[53,54]
**Entropy and information-theoretic measures**		
antropy_sample_entropy	Sample entropy (irregularity)	[50,55]
antropy_perm_entropy	Permutation entropy	[19,50]
antropy_spectral_entropy	Spectral entropy	[50,56]
**Phase-space geometry (delay embedding)**		
mean_1st_derivative	Mean local slope	[26]
var_1st_derivative	Variability of local slope	[26]
mean_2nd_derivative	Mean curvature	[26]
var_2nd_derivative	Curvature variability	[25,26]
**Linear-algebraic structure (SVD of embeddings)**		
sv_entropy	Entropy of singular values	[56,57]
sv_condition_number	Spectral conditioning	[58]
sv_spectral_flatness	Singular-value flatness	[59]
sv_effective_rank	Effective matrix rank	[57]

**Table 6:** Correlation summary for θ at window length W=100. The first row reports CatBoost predictions against ground truth on scaled windows. CatBoost results are presented only for scaled windows, since the models are trained on only scaled windows. All subsequent rows report ground truth vs. metric-derived surrogate values for the three data variants (scaled levels, unscaled levels, returns). The table highlights the results of our model as well as metrics highly correlated with the ground truth in bold.

Metric	Scaled Levels			Unscaled Levels			Returns		
	$\rho_{S}$	r	$R^{2}$	$\rho_{S}$	r	$R^{2}$	$\rho_{S}$	r	$R^{2}$
**CatBoost (model)**	**0.902**	**0.929**	**0.864**	–	–	–	–	–	–
hill_index	0.666	0.010	0.000	0.677	0.007	0.000	0.245	0.007	0.000
xi_pickands	0.426	0.253	0.064	0.397	0.246	0.061	0.159	0.128	0.016
xi_moment	0.488	0.377	0.142	0.488	0.361	0.131	0.070	0.066	0.004
tail_rate	0.043	0.031	0.001	0.272	0.175	0.031	0.272	0.175	0.031
skewness	0.008	0.007	0.000	0.007	0.007	0.000	−0.000	0.001	0.000
**kurtosis_excess**	**0.659**	**0.623**	**0.389**	**0.646**	**0.624**	**0.390**	**0.040**	**0.006**	**0.000**
hurst_h	−0.169	0.111	0.012	−0.184	0.093	0.009	–	–	–
hurst_catboost_ml	0.621	0.576	0.331	0.584	0.566	0.321	0.087	0.111	0.012
dfa_alpha	0.230	0.165	0.027	0.230	0.165	0.027	0.072	0.077	0.006
lag1_autocorr	0.000	0.004	0.000	−0.002	0.004	0.000	−0.001	0.004	0.000
ar1_phi	0.000	0.004	0.000	−0.002	0.004	0.000	−0.001	0.004	0.000
**theta_from_phi**	**0.842**	**0.895**	**0.800**	**0.842**	**0.895**	**0.800**	**0.027**	**0.134**	**0.018**
half_life_from_phi	0.842	0.138	0.019	0.842	0.138	0.019	−0.022	0.026	0.001
variance_ratio_q2	0.439	0.253	0.064	0.438	0.253	0.064	0.113	0.061	0.004
variance_ratio_q5	0.518	0.282	0.079	0.518	0.281	0.079	0.193	0.096	0.009
adf_stat	0.098	0.019	0.000	0.014	0.055	0.003	0.032	0.102	0.010
antropy_sample_entropy	0.181	0.231	0.053	0.213	0.233	0.054	0.060	0.008	0.000
antropy_perm_entropy	0.003	0.091	0.008	0.081	0.147	0.022	0.000	0.111	0.012
**antropy_spectral_entropy**	**0.773**	**0.802**	**0.644**	**0.807**	**0.792**	**0.628**	**0.155**	**0.268**	**0.072**
mean_1st_derivative	0.268	0.309	0.095	0.023	0.008	0.000	0.022	0.008	0.000
var_1st_derivative	0.238	0.165	0.027	0.012	0.007	0.000	0.012	0.007	0.000
mean_2nd_derivative	0.277	0.312	0.097	0.022	0.008	0.000	0.022	0.008	0.000
var_2nd_derivative	0.323	0.319	0.102	0.012	0.007	0.000	0.012	0.007	0.000
sv_entropy	0.327	0.396	0.157	0.751	0.811	0.659	−0.277	0.084	0.007
sv_condition_number	0.003	0.004	0.000	0.003	0.004	0.000	−0.000	0.004	0.000
sv_spectral_flatness	0.333	0.402	0.161	0.743	0.794	0.630	−0.275	0.084	0.007
sv_effective_rank	0.327	0.391	0.152	0.751	0.834	0.696	−0.277	0.077	0.006

**Table 7:** Correlation summary for α at window length W=100. The first row reports CatBoost predictions against ground truth on scaled windows. All subsequent rows report ground truth vs. metric-derived surrogate values for the three data variants (scaled levels, unscaled levels, returns). The table highlights the results of our model as well as metrics highly correlated with the ground truth in bold.

Metric	Scaled Levels			Unscaled Levels			Returns		
	$\rho_{S}$	r	$R^{2}$	$\rho_{S}$	r	$R^{2}$	$\rho_{S}$	r	$R^{2}$
**CatBoost (model)**	**0.938**	**0.937**	**0.878**	–	–	–	–	–	–
hill_index	−0.284	−0.285	0.081	−0.342	−0.342	0.117	−0.403	−0.404	0.163
xi_pickands	0.344	0.344	0.119	0.425	0.425	0.181	0.431	0.432	0.186
xi_moment	−0.034	−0.032	0.001	−0.000	−0.000	0.000	0.339	0.340	0.116
tail_rate	−0.163	−0.163	0.026	−0.163	−0.163	0.027	−0.163	−0.163	0.027
skewness	−0.010	−0.010	0.000	−0.012	−0.012	0.000	−0.004	−0.004	0.000
kurtosis_excess	0.336	0.338	0.114	0.427	0.427	0.182	0.532	0.534	0.285
hurst_h	0.604	0.356	0.127	0.603	0.378	0.143	–	–	–
hurst_catboost_ml	0.539	0.535	0.286	0.512	0.509	0.260	0.813	0.813	0.662
dfa_alpha	−0.359	−0.145	0.021	−0.359	−0.145	0.021	−0.321	−0.308	0.095
lag1_autocorr	−0.131	−0.003	0.000	−0.131	−0.003	0.000	−0.312	0.003	0.000
ar1_phi	−0.131	−0.003	0.000	−0.131	−0.003	0.000	−0.313	0.003	0.000
theta_from_phi	0.135	0.091	0.008	0.135	0.091	0.008	0.728	0.669	0.447
half_life_from_phi	−0.135	−0.138	0.019	−0.135	−0.138	0.019	−0.728	−0.035	0.001
variance_ratio_q2	−0.327	−0.491	0.241	−0.327	−0.491	0.241	−0.257	−0.442	0.195
variance_ratio_q5	−0.323	−0.483	0.234	−0.322	−0.484	0.234	−0.285	−0.439	0.193
adf_stat	0.288	0.186	0.034	0.316	0.187	0.035	0.234	0.111	0.012
**antropy_sample_entropy**	**−0.910**	**−0.909**	**0.827**	**−0.909**	**−0.908**	**0.825**	**−0.937**	**−0.937**	**0.878**
**antropy_perm_entropy**	**−0.841**	**−0.838**	**0.703**	**−0.865**	**−0.863**	**0.745**	**−0.766**	**−0.762**	**0.581**
antropy_spectral_entropy	0.058	0.058	0.003	−0.012	−0.011	0.000	0.051	0.059	0.004
**mean_1st_derivative**	**−0.785**	**−0.784**	**0.615**	**0.144**	**0.145**	**0.021**	**0.088**	**0.088**	**0.008**
var_1st_derivative	0.115	0.124	0.015	0.240	0.242	0.059	0.205	0.208	0.043
mean_2nd_derivative	−0.771	−0.770	0.592	0.110	0.111	0.012	0.071	0.072	0.005
var_2nd_derivative	0.109	0.118	0.014	0.211	0.213	0.045	0.181	0.184	0.034
sv_entropy	−0.384	−0.380	0.144	−0.303	−0.300	0.090	−0.164	−0.158	0.025
sv_condition_number	0.386	0.381	0.146	0.307	0.304	0.092	0.154	0.147	0.022
sv_spectral_flatness	−0.388	−0.384	0.148	−0.320	−0.316	0.100	−0.177	−0.169	0.029
sv_effective_rank	−0.384	−0.380	0.144	−0.303	−0.300	0.090	−0.164	−0.158	0.025

**Table 8:** Absolute counts per α category by period and asset.

Period	Asset	α = 2.0	α = 1.5	α = 1.0	α = 0.5	α = 0.05	Rows
1995–2004	AAPL	2297	1383	97	1	0	3778
	DJI	2954	801	22	1	0	3778
	GSPC	2982	759	37	0	0	3778
	MSFT	2560	1183	35	0	0	3778
2010–2024	AAPL	2325	1351	94	3	0	3773
	DJI	2472	1221	80	0	0	3773
	GSPC	2451	1281	41	0	0	3773
	MSFT	2518	1226	29	0	0	3773

**Table 9:** Absolute counts per θ category by period and asset.

Period	Asset	θ=32	θ=16	θ=8	θ=4	θ=2	$\theta =10^{-6}$	Rows
1995–2004	AAPL	171	333	531	540	715	1488	3778
	DJI	198	372	597	625	805	1181	3778
	GSPC	202	386	599	592	805	1194	3778
	MSFT	185	433	535	503	693	1429	3778
2010–2024	AAPL	116	354	534	575	833	1361	3773
	DJI	139	397	595	600	809	1233	3773
	GSPC	115	399	571	585	822	1281	3773
	MSFT	196	487	536	543	766	1245	3773

**Table 10:** Sunspots: absolute counts per α distribution category by period.

Period	Gaussian	Lévy	Observations
1985–2004	4805	2500	7305
2005–2024	3730	3575	7305

**Table 11:** Sunspots: absolute counts per θ mean-reversion category by period.

Period	No Mean Reversion	Mean Reversion	Observations
1985–2004	2216	5089	7305
2005–2024	3311	3994	7305

**Table 12:** Absolute counts per α category by period and variable (all Austrian locations pooled; 50-day windows, step 1).

Period	Variable	α=2.0	α=1.5	α=1.0	α=0.5	α=0.05	Rows
1985–2004	ALLSKY_SFC_SW_DWN	252,661	201,027	69,658	2,605	9	525,960
	CLRSKY_SFC_SW_DWN	310,660	194,413	20,817	40	30	525,960
	PRECTOTCORR	368	174,242	340,834	10,510	6	525,960
	PS	442,210	83,322	428	0	0	525,960
	RH2M	309,072	192,538	24,306	44	0	525,960
	T2MDEW	419,656	105,534	770	0	0	525,960
	T2M_RANGE	354,956	147,121	23,883	0	0	525,960
	TOA_SW_DWN	0	4490	49,237	221,661	250,572	525,960
	WS10M	230,010	275,010	20,940	0	0	525,960
	WS10M_MAX	197,802	287,956	40,202	0	0	525,960
2005–2025	ALLSKY_SFC_SW_DWN	267,713	204,414	53,833	0	0	525,960
	CLRSKY_SFC_SW_DWN	395,689	125,553	4715	3	0	525,960
	PRECTOTCORR	133	173,071	341,732	11,021	3	525,960
	PS	431,877	93,619	464	0	0	525,960
	RH2M	307,729	191,274	26,929	28	0	525,960
	T2MDEW	407,164	117,698	1098	0	0	525,960
	T2M_RANGE	355,124	147,867	22,969	0	0	525,960
	TOA_SW_DWN	0	4123	50,873	220,386	250,578	525,960
	WS10M	218,228	287,997	19,735	0	0	525,960
	WS10M_MAX	179,446	304,033	42,481	0	0	525,960

**Table 13:** Absolute counts per θ category by period and variable (all Austrian locations pooled; 50-day windows, step 1).

Period	Variable	θ=32	θ=16	θ=8	θ=4	θ=2	$\theta \;=\;10^{-6}$	Rows
1985–2004	ALLSKY_SFC_SW_DWN	456,012	37,174	14,292	6148	3456	8878	525,960
	CLRSKY_SFC_SW_DWN	90,355	35,795	40,424	50,499	150,038	158,849	525,960
	PRECTOTCORR	468,123	18,560	3228	628	70	35,351	525,960
	PS	232,784	164,141	61,575	24,173	14,165	29,122	525,960
	RH2M	431,376	53,260	16,427	6073	3040	15,784	525,960
	T2MDEW	169,764	135,708	84,280	42,721	31,348	62,139	525,960
	T2M_RANGE	513,624	9403	1403	507	304	719	525,960
	TOA_SW_DWN	0	0	33,038	47,461	143,285	302,176	525,960
	WS10M	510,129	11,263	1627	635	297	2009	525,960
	WS10M_MAX	508,478	11,801	1908	751	398	2624	525,960
2005–2025	ALLSKY_SFC_SW_DWN	434,606	47,345	18,824	8867	5184	11,134	525,960
	CLRSKY_SFC_SW_DWN	77,316	30,959	35,538	50,434	143,284	188,429	525,960
	PRECTOTCORR	472,260	15,461	2829	652	75	34,683	525,960
	PS	232,180	163,224	63,911	22,791	13,595	30,259	525,960
	RH2M	437,683	49,014	15,902	5816	2776	14,769	525,960
	T2MDEW	159,468	144,860	80,681	42,107	31,742	67,102	525,960
	T2M_RANGE	513,303	8990	1646	710	370	941	525,960
	TOA_SW_DWN	0	0	33,775	46,879	142,835	302,471	525,960
	WS10M	515,611	6686	989	479	292	1903	525,960
	WS10M_MAX	511,320	9902	1550	601	402	2185	525,960

**Figure A1:**
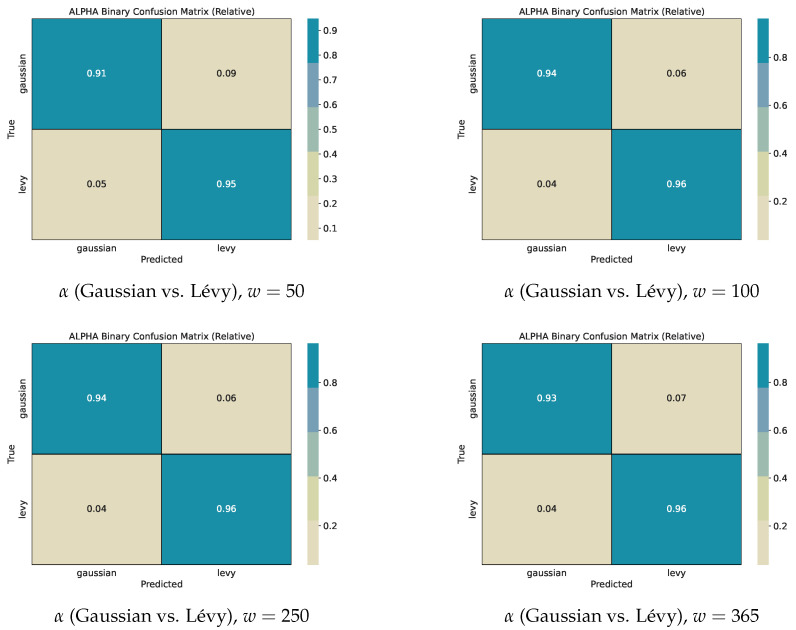
Binary confusion matrices for α classification (Gaussian vs. Lévy) across window sizes. Panels show row-normalized confusion matrices for w∈{50,100,250,365}.

**Figure A2:**
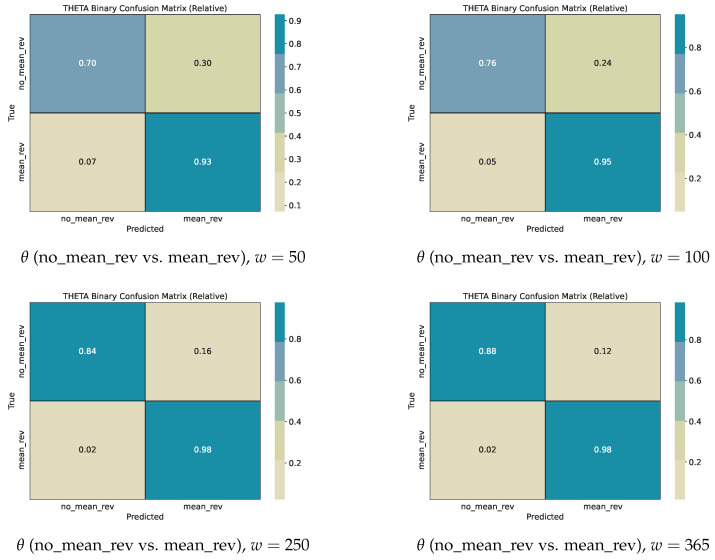
Binary confusion matrices for θ classification (no mean reversion vs. mean reversion) across window sizes. Panels show row-normalized confusion matrices for w∈{50,100,250,365}.

## Data Availability

Data openly available through APIs as shown in our corresponding repository at https://github.com/Raubkatz/HeavyTailsMeanReversion (accessed on 15 December 2025).

## Appendix A. Extended Validation: Binary Tasks (Gaussian vs. Non-Gaussian; Strong vs. Weak Mean Reversion)

To complement the multi-class evaluation, we re-cast both problems as *binary* decisions and test the same trained models without retraining: (i) **Gaussian vs. Lévy** for α (where the positive class “Gaussian” corresponds to α=2.0, and “Lévy” aggregates α∈{1.5, 1.0, 0.5, 0.05}) and (ii) **strong mean reversion vs. weak/no mean reversion** for θ (where “mean_rev” aggregates θ∈{2, 4, 8, 16, 32} and “no_mean_rev” corresponds to $\theta \;=\;10^{-6}$). These binary diagnostics are practically useful: in many applications it suffices to know whether a window is *approximately Gaussian* (vs. heavy-tailed) or *strongly* mean-reverting (vs. weak), and such coarse screening can be more robust than fine-grained multi-class labeling.

Across window sizes, the **Gaussian vs. Lévy** task is learned very effectively. At w=50, accuracy is 0.94 (weighted $F_{1}\;=\;0.94$), with high recall for the Gaussian class (0.91) and excellent precision/recall for the Lévy class (0.98/0.95). As the window grows to w=100 and w=250, performance tightens further (accuracy 0.957–0.959), reflecting the increased chance to observe tail behavior and volatility clustering within a longer segment. At w=365, performance remains at the same high level (accuracy 0.957, weighted $F_{1}\;=\;0.957$), confirming that the Gaussian vs. Lévy separation saturates once sufficient window length is available.

For **mean reversion**, the binary split is more challenging at smaller windows but improves substantially with *w*: at w=50, we attain accuracy 0.889 (weighted $F_{1}\;=\;0.890$) with lower recall for no_mean_rev (0.70)—reasonable given that weak reversion is expressed by an *absence* of pull, which is harder to certify in short segments. By w=100, the scores rise (accuracy 0.919), and at w=250, the classifier reaches accuracy 0.954 with balanced precision/recall for both classes (≈0.86–0.98). At w=365, the binary θ classifier improves further, reaching accuracy 0.967 (weighted $F_{1}\;=\;0.967$), with high recall for both no_mean_rev (0.88) and mean_rev (0.98).

In short, longer windows sharpen both binary tasks, but even w=50 already delivers strong Gaussian screening and useful mean-reversion flags. Figure A1 and Figure A2 collect all eight binary confusion matrices.

We mirror the formatting of the multi-class section: for each window size, α (left) and θ (right) reports are shown side by side.

Interpretation (binary).

For α, the classifier is a reliable *Gaussian screener*: already at w=50, it distinguishes Gaussian windows from heavy-tailed alternatives with ≈94% accuracy and balanced macro-averages, and it improves slightly with *w*. This indicates that the window-level complexity features capture distributional shape robustly, with longer windows providing more opportunities to observe tail events and intermittency. For θ, the binary split cleanly separates “presence” vs. “absence” of mean reversion by w=250 (accuracy ≈ 95%); at small *w*, weak reversion is harder to verify (lower recall for no_mean_rev), which is consistent with the temporal nature of reversion signals. Practically, these binary tests validate that the trained models can deliver crisp, interpretable flags (Gaussian vs. Lévy; strong vs. weak reversion) that align with the most consequential regime distinctions in downstream analyses.

**Table A1:** Binary reports for **50-point** windows.

α: Gaussian vs. Lévy				
Class	Prec.	Recall	F1-Score	Support
gaussian	0.81	0.91	0.86	948,000
levy	0.98	0.95	0.96	3,792,000
Accuracy = 0.9399, Weighted F1-Score = 0.9411				
**θ: no_mean_rev vs. mean_rev**				
Class	Prec.	Recall	F1-Score	Support
no_mean_rev	0.66	0.70	0.68	790,000
mean_rev	0.94	0.93	0.93	3,950,000
Accuracy = 0.8885, Weighted F1-Score = 0.8898				

**Table A2:** Binary reports for **100-point** windows.

α: Gaussian vs. Lévy				
Class	Prec.	Recall	F1-Score	Support
gaussian	0.86	0.94	0.90	468,000
levy	0.98	0.96	0.97	1,872,000
Accuracy = 0.9569, Weighted F1 = 0.9576				
**θ: no_mean_rev vs. mean_rev**				
Class	Prec.	Recall	F1-Score	Support
no_mean_rev	0.76	0.76	0.76	390,000
mean_rev	0.95	0.95	0.95	1,950,000
Accuracy = 0.9190, Weighted F1-Score = 0.9189				

**Table A3:** Binary reports for **250-point** windows.

α: Gaussian vs. Lévy				
Class	Prec.	Recall	F1-Score	Support
gaussian	0.87	0.94	0.90	180,000
levy	0.98	0.96	0.97	720,000
Accuracy = 0.9590, Weighted F1-Score = 0.9595				
**θ: no_mean_rev vs. mean_rev**				
Class	Prec.	Recall	F1-Score	Support
no_mean_rev	0.88	0.84	0.86	150,000
mean_rev	0.97	0.98	0.97	750,000
Accuracy = 0.9540, Weighted F1-Score = 0.9537				

**Table A4:** Binary reports for **365-point** windows.

α: Gaussian vs. Lévy				
Class	Prec.	Recall	F1-Score	Support
gaussian	0.86	0.93	0.90	108,000
levy	0.98	0.96	0.97	432,000
Accuracy = 0.9567, Weighted F1-Score = 0.9573				
**θ: no_mean_rev vs. mean_rev**				
Class	Prec.	Recall	F1-Score	Support
no_mean_rev	0.92	0.88	0.90	90,000
mean_rev	0.98	0.98	0.98	450,000
Accuracy = 0.9670, Weighted F1-Score = 0.9667				

## Appendix B. Statistical and Complexity Metrics

This appendix provides concise descriptions of all statistical and complexity-based metrics employed in the correlation analyses. The metrics are grouped by conceptual category, reflecting the different aspects of time-series structure they aim to capture, including tail behavior, mean reversion, scaling properties, entropy, geometric complexity, and linear-algebraic structure.

All metrics listed here are summarized in Table 5. In the main analysis, these metrics are evaluated on scaled series, unscaled series, and increment (return) series, and their outputs are compared against ground-truth parameters and machine learning-based estimates using correlation-based criteria.

Unless stated otherwise, metrics are applied to individual sliding windows of fixed length, and multiple realizations or parameterizations of conceptually similar quantities (e.g., Hurst-type or variance-ratio measures) are included to assess robustness across methodological choices.

### Appendix B.1. Tail Behavior and Distributional Shape Metrics

This subsection defines the tail-related estimators used in the metric library. Throughout, we compute tail statistics on a nonnegative magnitude series derived from the original window, in order to avoid sign cancellations and to obtain a one-sided tail object. Unless stated otherwise, the magnitude series is defined as the absolute deviation from the sample median. The full notation is summarized at the end of this subsection.

- **Hill index (****hill_index****).** Let $Y_{1},\ldots ,Y_{n}$ be nonnegative magnitudes and let $Y_{\left(1\right)}\leq \ldots \leq Y_{\left(n\right)}$ denote the order statistics. For a chosen tail size *k* with 1<k<n, the Hill estimator targets the Pareto-type tail index and is given by
  (A1) $${\hat{\xi }}_{H}\left(k\right)\;=\;\frac{1}{k}\sum_{j=1}^{k}\log\;\left(\frac{Y_{\left(n-j+1\right)}}{Y_{\left(n-k\right)}}\right),\;(\mathrm{requires}\;Y_{\left(n-k\right)}>0).$$
  For regularly varying tails, ξ>0 corresponds to heavy tails (polynomial decay); larger $\hat{\xi }$ indicates heavier tails (slower decay). The choice of *k* controls the bias–variance trade-off [8,41,76].
- **Pickands estimator (****xi_pickands****).** For *k* such that 4k<n, Pickands’ estimator uses ratios of spacings between upper-order statistics:
  (A2) $${\hat{\xi }}_{P}\left(k\right)\;=\;\frac{1}{\log2}\;\log\;\left(\frac{Y_{\left(n-k\right)}-Y_{\left(n-2k\right)}}{Y_{\left(n-2k\right)}-Y_{\left(n-4k\right)}}\right),$$
  provided the numerator and denominator are positive. This is a classical estimator for the extreme value index ξ under standard EVT conditions [42,76].
- **Moment estimator (****xi_moment****).** The Dekkers–Einmahl–de Haan moment estimator combines first and second log-moments of tail ratios to stabilize estimation. Let
  (A3) $$M_{1}\left(k\right)\;=\;\frac{1}{k}\sum_{j=1}^{k}\log\;\left(\frac{Y_{\left(n-j+1\right)}}{Y_{\left(n-k\right)}}\right),\;M_{2}\left(k\right)\;=\;\frac{1}{k}\sum_{j=1}^{k}{\left[\log\;\left(\frac{Y_{\left(n-j+1\right)}}{Y_{\left(n-k\right)}}\right)\right]}^{2}.$$
  Then the moment estimator is
  (A4) $${\hat{\xi }}_{M}\left(k\right)\;=\;M_{1}\left(k\right)\;+\;1\;-\;\frac{1}{2}{\left(1-\frac{M_{1}{\left(k\right)}^{2}}{M_{2}\left(k\right)}\right)}^{-1},$$
  whenever $M_{2}\left(k\right)>0$ and the denominator is well defined [43,76].
- **Tail exceedance rate (****tail_rate****).** Let $Q_{Y}\left(q\right)$ denote the empirical *q*-quantile of $\left\{Y_{i}\right\}$. The exceedance rate is the empirical tail mass above this threshold:
  (A5) $${\hat{p}}_{q}\;=\;\frac{1}{n}\sum_{i=1}^{n}1\left\{Y_{i}>Q_{Y}\left(q\right)\right\}.$$
  For continuous data and standard quantile definitions, ${\hat{p}}_{q}$ is close to 1−q by construction; in finite samples, with ties or discretization, it becomes a simple proxy that can still vary across windows [44].
- **Skewness (****skewness****).** Skewness measures distributional asymmetry via the standardized third central moment:
  (A6) $$\gamma_{1}\;=\;\frac{\mu_{3}}{\sigma^{3}},\;\mu_{3}=E\;\left[{\left(X-\mu \right)}^{3}\right],\;\;\sigma^{2}=E\;\left[{\left(X-\mu \right)}^{2}\right].$$
  In practice, sample estimators replace (μ,σ) by sample mean and sample standard deviation (possibly with bias corrections, depending on the software implementation) [45].
- **Excess kurtosis (****kurtosis_excess****).** Excess kurtosis measures tail weight and peakedness relative to the Gaussian baseline:
  (A7) $$\gamma_{2}\;=\;\frac{\mu_{4}}{\sigma^{4}}-3,\;\mu_{4}=E\;\left[{\left(X-\mu \right)}^{4}\right].$$
  As for skewness, empirical estimators vary by bias-correction conventions. The metric uses the standard “excess” definition (subtracting 3) [45].

Notation (used in this subsection).

Let $X_{1},\ldots ,X_{n}$ denote the original series values within one time window. We define a nonnegative magnitude series

(A8) $$Y_{i}\;=\;\left|X_{i}-\mathrm{median}\left(X_{1},\ldots ,X_{n}\right)\right|,\;i=1,\ldots ,n,$$

and let $Y_{\left(1\right)}\leq \ldots \leq Y_{\left(n\right)}$ denote its order statistics. The integer *k* denotes the number of upper-order statistics used for tail estimation. Indicator functions are written as 1{·}. “ξ” denotes the extreme value index. “Excess kurtosis” is defined as kurtosis minus 3. “Quantile” refers to the empirical quantile computed on the current window. “Returns” refer to first differences $R_{t}=X_{t}-X_{t-1}$ when applied to increment series. “Scaled” and “unscaled” refer to the two data representations used in the main experiments. “Returns” metrics are computed on the increment series derived from the unscaled-level windows, as described in the main text.

### Appendix B.2. Mean Reversion, Memory, and Scaling Properties

This group contains metrics that quantify temporal dependence, persistence, and scaling behavior. In our setting, these properties are closely related to the mean-reversion parameter θ of the Ornstein–Uhlenbeck (OU) process and its discrete-time AR(1) approximation. In the empirical analysis, all metrics are evaluated consistently on (i) scaled windows, (ii) unscaled windows, and (iii) the corresponding return (increment) windows.

Notation.

Let ${\left\{x_{t}\right\}}_{t=1}^{n}$ denote a window of length *n*. Define increments (returns) $r_{t}:=x_{t}-x_{t-1}$ for t=2,…,n. Let $\bar{x}:=\frac{1}{n}\sum_{t=1}^{n}x_{t}$. For lag-*k* sample autocovariance and autocorrelation, we use

(A9) $$\begin{array}{cc} \hat{\gamma }\left(k\right) & :=\frac{1}{n-k}\sum_{t=k+1}^{n}\left(x_{t}-\bar{x}\right)\left(x_{t-k}-\bar{x}\right), \end{array}$$

(A10) $$\begin{array}{cc} \hat{\rho }\left(k\right) & :=\frac{\hat{\gamma }\left(k\right)}{\hat{\gamma }\left(0\right)}. \end{array}$$

- **Hurst exponent (****hurst_h****).** The Hurst exponent H∈(0,1) summarizes scaling and persistence. A common definition is based on self-similar scaling of fluctuations, e.g., for a process with stationary increments
  (A11) $$\mathrm{Var}\;\left(X(t+\tau )-X(t)\right)\;\propto \;\tau^{2H}.$$
  Heuristically, H<0.5 indicates anti-persistence (negative dependence of increments), H≈0.5 indicates behavior close to an uncorrelated random walk, and H>0.5 indicates persistence [46]. In our implementation, the hurst package is used (function compute_Hc) and the estimator configuration depends on whether we analyze levels or increments (e.g., kind=‘random_walk’ for level-like data and kind=‘change’ for increments) [47].
- **Machine learning-based Hurst proxy (****hurst_catboost_ml****).** This is a learned estimator of *H* produced by a pre-trained CatBoost model operating directly on windows. It provides a data-driven alternative to classical estimators while keeping the same target quantity (*H*). Methodologically, it follows gradient-boosted decision trees as implemented in CatBoost [39].
- **Detrended fluctuation analysis exponent (****dfa_alpha****).** DFA estimates a scaling exponent $\alpha_{\mathrm{DFA}}$ from the relationship between the fluctuation function F(s) and the time scale *s*. Starting from the demeaned series, define the integrated profile
  (A12) $$Y\left(k\right):=\sum_{t=1}^{k}\left(x_{t}-\bar{x}\right),\;k=1,\ldots ,n.$$
  For a given segment length *s*, divide *Y* into $N_{s}:=\left\lfloor n/s\right\rfloor$ non-overlapping segments. For each segment $\nu =1,\ldots ,N_{s}$, fit a polynomial trend ${\hat{Y}}_{\nu ,s}\left(j\right)$ to the local profile values $Y\left((\nu -1)s+j\right)$ for j=1,…,s, and compute the mean squared deviation within the segment,
  (A13) $$F_{\nu }^{2}\left(s\right):=\frac{1}{s}\sum_{j=1}^{s}{\left(Y\left((\nu -1)s+j\right)-{\hat{Y}}_{\nu ,s}\left(j\right)\right)}^{2}.$$
  The fluctuation function is obtained by averaging over all segments and taking the square root,
  (A14) $$F\left(s\right):=\sqrt{\frac{1}{N_{s}}\sum_{\nu =1}^{N_{s}}F_{\nu }^{2}\left(s\right)}.$$
  If F(s) follows a power-law relationship across scales,
  (A15) $$F\left(s\right)\;\propto \;s^{\alpha_{\mathrm{DFA}}},$$
  then $\alpha_{\mathrm{DFA}}$ is defined as the DFA exponent [48,49]. In the implementation, antropy.detrended_fluctuation is used as the primary routine, with nolds.dfa as a fallback if required [50,77].
- **Lag-1 autocorrelation (****lag1_autocorr****).** This metric is the empirical lag-1 autocorrelation
  (A16) $$\hat{\rho }\left(1\right)\;=\;\frac{\hat{\gamma }\left(1\right)}{\hat{\gamma }\left(0\right)}.$$
  For OU-like dynamics (mean reversion), smaller $\hat{\rho }\left(1\right)$ indicates faster reversion in discrete time [51].
- **AR(1) persistence parameter (****ar1_phi****).** We estimate the AR(1) coefficient ϕ by ordinary least squares in
  (A17) $$x_{t}=c+\phi x_{t-1}+\varepsilon_{t},$$
  which yields the ratio [51]:
  (A18) $$\hat{\phi }\;=\;\frac{\mathrm{Cov}(x_{t-1},x_{t})}{\mathrm{Var}(x_{t-1})}.$$
- **OU mean-reversion rate proxy (****theta_from_phi****).** For the OU SDE
  (A19) $$dX_{t}=\theta \left(\mu -X_{t}\right)\;dt+\sigma \;dW_{t},$$
  the exact discrete-time transition at step Δt implies an AR(1)-type dependence with
  (A20) $$\phi \;=\;e^{-\theta \Delta t}.$$
  Therefore, a window-wise proxy for θ is
  (A21) $$\hat{\theta }\;=\;-\frac{\ln(\hat{\phi })}{\Delta t},$$
  valid when $\hat{\phi }\in \left(0,1\right)$ [1].
- **Mean-reversion half-life (****half_life_from_phi****).** Under OU dynamics, the half-life satisfies
  (A22) $$t_{1/2}\;=\;\frac{\ln(2)}{\theta },$$
  and using the proxy above gives ${\hat{t}}_{1/2}:=\ln2/\hat{\theta }$ (when $\hat{\theta }>0$) [1].
- **Variance-ratio tests (****variance_ratio_q2****,** **variance_ratio_q5****).** Let $\left\{r_{t}\right\}$ be increments. For an aggregation horizon q≥2, define aggregated increments $R_{t}^{\left(q\right)}:=\sum_{i=0}^{q-1}r_{t-i}$ (for indices where this is defined). The variance ratio is
  (A23) $$\mathrm{VR}\left(q\right)\;:=\;\frac{\mathrm{Var}(R_{t}^{\left(q\right)})}{q\;\mathrm{Var}(r_{t})}.$$
  For an uncorrelated random walk, VR(q)≈1. Values VR(q)<1 indicate negative serial correlation, while VR(q)>1 indicates positive serial correlation [52].
- **Augmented Dickey–Fuller statistic (****adf_stat****).** The ADF test fits the regression
  (A24) $$\Delta x_{t}=\alpha +\beta t+\gamma x_{t-1}+\sum_{i=1}^{p}\delta_{i}\;\Delta x_{t-i}+\varepsilon_{t},$$
  and tests the null hypothesis of a unit root via $H_{0}:\gamma =0$. The reported adf_stat is the test statistic for γ. More negative values provide stronger evidence against the unit-root null in favor of stationarity [53]. In our scripts, the statistic is computed via statsmodels.tsa.stattools.adfuller [54].

Notation summary.

A windowed time series is $x=(x_{1},\ldots ,x_{N})$. For delay embedding, τ is the delay, *d* is the embedding dimension, and $y_{t}=\left(x_{t},x_{t+\tau },\ldots ,x_{t+(d-1)\tau }\right)$ for t=1,…,T with T=N−(d−1)τ. Finite-difference derivatives are ${\dot{y}}_{t}\approx y_{t+1}-y_{t}$ and ${\ddot{y}}_{t}\approx {\dot{y}}_{t+1}-{\dot{y}}_{t}$. For SVD metrics, $\sigma_{1}\geq \ldots \geq \sigma_{r}>0$ are singular values and $p_{i}=\sigma_{i}/\sum_{j}\sigma_{j}$.

### Appendix B.3. Entropy and Information-Theoretic Complexity

Let $x=(x_{1},\ldots ,x_{N})$ denote a real-valued time series (window). Throughout, we assume missing values have been removed and that *N* is sufficiently large for the estimator in question.

- **Sample entropy (****antropy_sample_entropy****).** Sample entropy (SampEn) measures the *negative logarithm* of the conditional probability that two sequences which are similar for *m* consecutive samples remain similar at the next sample, excluding self-matches [55]. Fix an embedding dimension m∈N and a tolerance r>0. Define the length-*m* template vectors
  (A25) $$u_{i}^{\left(m\right)}=\left(x_{i},x_{i+1},\ldots ,x_{i+m-1}\right),\;i=1,\ldots ,N-m+1.$$
  Using a norm ∥·∥ (commonly the Chebyshev norm ${∥a∥}_{\infty }=\max_{k}\left|a_{k}\right|$), define the counts of matches
  (A26) $$\begin{array}{cc} B_{i}^{\left(m\right)}\left(r\right) & =\#\left\{j\in \left\{1,\ldots ,N-m+1\right\}\setminus \left\{i\right\}:∥u_{i}^{\left(m\right)}-u_{j}^{\left(m\right)}∥\leq r\right\}, \end{array}$$
  (A27) $$\begin{array}{cc} A_{i}^{\left(m\right)}\left(r\right) & =\#\left\{j\in \left\{1,\ldots ,N-m\right\}\setminus \left\{i\right\}:∥u_{i}^{\left(m+1\right)}-u_{j}^{\left(m+1\right)}∥\leq r\right\}, \end{array}$$
  where $u_{i}^{\left(m+1\right)}=\left(x_{i},x_{i+1},\ldots ,x_{i+m}\right)$ for i=1,…,N−m. Let
  (A28) $$B^{\left(m\right)}\left(r\right)=\sum_{i=1}^{N-m+1}B_{i}^{\left(m\right)}\left(r\right),\;A^{\left(m\right)}\left(r\right)=\sum_{i=1}^{N-m}A_{i}^{\left(m\right)}\left(r\right).$$
  Then SampEn is
  (A29) $$\mathrm{SampEn}\left(m,r;x\right)=-\ln\;\left(\frac{A^{\left(m\right)}\left(r\right)}{B^{\left(m\right)}\left(r\right)}\right),$$
  provided $A^{\left(m\right)}\left(r\right)>0$ and $B^{\left(m\right)}\left(r\right)>0$. Larger SampEn indicates greater irregularity/lower predictability. The implementation used here follows the AntroPy API (parameters include order = m and a distance metric) [78].
- **Permutation entropy (****antropy_perm_entropy****).** Permutation entropy (PermEn) quantifies complexity via ordinal patterns of length *m* with delay τ [19]. For each i=1,…,N−(m−1)τ, form the embedded vector
  (A30) $$v_{i}=\left(x_{i},x_{i+\tau },\ldots ,x_{i+(m-1)\tau }\right).$$
  Let $\pi (v_{i})$ denote the permutation (ordinal pattern) that sorts the components of $v_{i}$ in increasing order (ties are handled by a fixed convention in software implementations). Over all m! possible permutations, define empirical probabilities
  (A31) $$p\left(\pi \right)=\frac{1}{N-(m-1)\tau }\sum_{i=1}^{N-(m-1)\tau }1\left\{\pi \left(v_{i}\right)=\pi \right\}.$$
  Permutation entropy is the Shannon entropy of this distribution:
  (A32) $$H_{\mathrm{perm}}\left(m,\tau ;x\right)=-\sum_{\pi }p\left(\pi \right)\;\log p\left(\pi \right).$$
  A common normalization is
  (A33) $$H_{\mathrm{perm}}^{\mathrm{norm}}\left(m,\tau ;x\right)=\frac{H_{\mathrm{perm}}\left(m,\tau ;x\right)}{\log(m!)}\in \left[0,1\right],$$
  with higher values indicating richer ordinal dynamics (more uniformly distributed patterns). The implementation follows the AntroPy’s perm_entropy interface and constraints on feasible (m,τ) for finite *N* [79].
- **Spectral entropy (****antropy_spectral_entropy****).** Spectral entropy measures the dispersion of power across frequencies by applying Shannon entropy to a *normalized* power spectral density (PSD) [56]. Let $P(f_{k})\geq 0$ be a discrete PSD estimate over frequency bins $f_{k}$. Define a probability mass function
  (A34) $$p_{k}=\frac{P(f_{k})}{\sum_{j}P\left(f_{j}\right)}.$$
  The spectral entropy (in bits) is
  (A35) $$H_{\mathrm{spec}}\left(x\right)=-\sum_{k}p_{k}\log_{2}\left(p_{k}\right),$$
  and a typical normalization is $H_{\mathrm{spec}}^{\mathrm{norm}}=H_{\mathrm{spec}}/\log_{2}K\in \left[0,1\right]$ for *K* bins. In our analysis pipeline, PSD estimation uses either Welch’s method or an FFT-based estimate depending on window length; this matches AntroPy’s API (sf, method, normalize) [80]. Higher spectral entropy indicates a more broadband spectrum (less concentration in narrow frequency bands).

### Appendix B.4. Phase-Space Geometry and Local Dynamics

These metrics are computed after reconstructing a state-space trajectory from a scalar time series via delay-coordinate embedding [81,82]. Fix an embedding dimension *d* and delay τ. Define embedded vectors

(A36) $$y_{t}=\left(x_{t},x_{t+\tau },\ldots ,x_{t+(d-1)\tau }\right)\in R^{d},\;t=1,\ldots ,T,$$

where T=N−(d−1)τ.

We treat ${\left\{y_{t}\right\}}_{t=1}^{T}$ as a discrete curve and compute finite-difference derivatives with respect to the discrete index *t*. Let

(A37) $${\dot{y}}_{t}\approx y_{t+1}-y_{t},\;{\ddot{y}}_{t}\approx {\dot{y}}_{t+1}-{\dot{y}}_{t},$$

with suitable endpoint handling (software uses standard numerical gradients).

- **Mean first-derivative norm (****mean_1st_derivative****).** Define speeds $s_{t}={∥{\dot{y}}_{t}∥}_{2}$. The metric is
  (A38) $$\mu_{s}=\frac{1}{T-1}\sum_{t=1}^{T-1}s_{t}.$$
  Larger values indicate faster local motion in reconstructed state space (greater short-scale variability in the embedded trajectory).
- **Variance of first-derivative norm (****var_1st_derivative****).** Using $s_{t}$ as above,
  (A39) $$\mathrm{Var}\left(s\right)=\frac{1}{T-1}\sum_{t=1}^{T-1}{\left(s_{t}-\mu_{s}\right)}^{2}.$$
  Higher values indicate stronger heterogeneity of local “speeds” along the trajectory.
- **Mean second-derivative norm (****mean_2nd_derivative****).** Define curvature-proxy magnitudes $c_{t}={∥{\ddot{y}}_{t}∥}_{2}$. The metric is
  (A40) $$\mu_{c}=\frac{1}{T-2}\sum_{t=1}^{T-2}c_{t}.$$
  Larger values indicate stronger changes in direction/speed in the reconstructed trajectory (more pronounced local bending).
- **Variance of second-derivative norm (****var_2nd_derivative****).**
  (A41) $$\mathrm{Var}\left(c\right)=\frac{1}{T-2}\sum_{t=1}^{T-2}{\left(c_{t}-\mu_{c}\right)}^{2}.$$
  Higher values indicate greater variability in local curvature, consistent with more heterogeneous local dynamics in the embedding [81,82].

**Remark (role in this work).** These are *feature-style* geometric summaries computed on short windows; they are not intended as invariant dynamical system estimators. Their value in our workflow is empirical: correlation against known OU parameters across scaled/unscaled/return variants.

### Appendix B.5. Linear-Algebraic Structure of Embedded Dynamics (SVD Metrics)

To connect the time-series structure to linear-algebraic summaries, we apply singular-value decomposition (SVD) to a delay-embedded matrix. Let $M\in R^{T\times d}$ be the embedding matrix whose rows are $y_{t}^{⊤}$. Optionally, we use a square Gram matrix $G=M^{⊤}M$ (or $MM^{⊤}$). If *G* is used, its eigenvalues $\left\{\lambda_{i}\left(G\right)\right\}$ satisfy $\lambda_{i}\left(G\right)=\sigma_{i}{\left(M\right)}^{2}$, so the corresponding singular values of *M* are obtained as $\sigma_{i}\left(M\right)=\sqrt{\lambda_{i}\left(G\right)}$.

Let $\sigma_{1}\geq \sigma_{2}\geq \ldots \geq \sigma_{r}>0$ denote the (positive) singular values of *M* (with r=rank(M)).

- **Singular-value entropy (****sv_entropy****).** Define normalized singular-value weights
  (A42) $$p_{i}=\frac{\sigma_{i}}{\sum_{j=1}^{r}\sigma_{j}}.$$
  The singular-value entropy is the Shannon entropy of $\left\{p_{i}\right\}$:
  (A43) $$H_{\mathrm{SV}}=-\sum_{i=1}^{r}p_{i}\log p_{i},$$
  with higher values indicating a more even spectrum (less dominance by a small number of modes) [56].
- **Condition number (****sv_condition_number****).** The spectral condition number is
  (A44) $$\kappa =\frac{\sigma_{1}}{\sigma_{r}}.$$
  Large κ indicates strong anisotropy/near-low-rank structure and numerical ill-conditioning [58].
- **Spectral flatness (****sv_spectral_flatness****).** Spectral flatness (also known as Wiener entropy in signal-processing contexts) is the ratio of the geometric mean to the arithmetic mean of positive spectrum values [83,84]:
  (A45) $$\mathrm{SFM}=\frac{{\left(\prod_{i=1}^{r}\sigma_{i}\right)}^{1/r}}{\frac{1}{r}\sum_{i=1}^{r}\sigma_{i}}.$$
  Values close to 1 correspond to a flatter (more uniform) singular-value spectrum; small values indicate strong dominance of a few singular values.
- **Effective rank (****sv_effective_rank****).** Effective rank provides a smooth intrinsic-dimensionality proxy based on entropy of the normalized spectrum [57]. With $p_{i}$ as above,
  (A46) $$r_{\mathrm{eff}}=\exp\;\left(-\sum_{i=1}^{r}p_{i}\log p_{i}\right)=\exp\left(H_{\mathrm{SV}}\right).$$
  If one singular value dominates, then $r_{\mathrm{eff}}\approx 1$; if the spectrum is uniform across *r* modes, then $r_{\mathrm{eff}}\approx r$.

Notation summary.

A windowed time series is $x=(x_{1},\ldots ,x_{N})$. For delay embedding, τ is the delay, *d* is the embedding dimension, and $y_{t}=\left(x_{t},x_{t+\tau },\ldots ,x_{t+(d-1)\tau }\right)$ for t=1,…,T with T=N−(d−1)τ. Finite-difference derivatives are ${\dot{y}}_{t}\approx y_{t+1}-y_{t}$ and ${\ddot{y}}_{t}\approx {\dot{y}}_{t+1}-{\dot{y}}_{t}$. For SVD metrics, $\sigma_{1}\geq \ldots \geq \sigma_{r}>0$ are singular values and $p_{i}=\sigma_{i}/\sum_{j}\sigma_{j}$.

## Appendix C. Additional Results and Correlation Tests

This appendix provides additional correlation summaries for window lengths other than the main configuration discussed in Section 6.3. The tables replicate the same evaluation protocol: for each window metric, we report Spearman rank correlation $\rho_{S}$, Pearson correlation *r*, and linear recalibration $R^{2}$ between the metric-derived surrogate and the ground-truth label, evaluated on (i) scaled levels, (ii) unscaled levels, and (iii) returns. The first row in each table reports CatBoost predictions against ground truth on scaled windows. The purpose of this appendix is to assess how stable the observed patterns are as a function of window length.

### Appendix C.1. Additional Results for **α**

Table A5, Table A6 and Table A7 report the correlation summaries for α at W∈{50, 250, 365}. These results complement Table 7 in Section 6.3 and allow a comparison of metric rankings and preprocessing sensitivity across window lengths.

At W=50 (Table A5), CatBoost remains strongly correlated with the ground truth on scaled levels ($\rho_{S}=0.934$, r=0.932, $R^{2}=0.869$). Among classical metrics on scaled levels, the strongest predictors remain the entropy and derivative family: antropy_sample_entropy ($\rho_{S}=-0.897$, r=−0.896, $R^{2}=0.803$), antropy_perm_entropy ($\rho_{S}=-0.812$, r=−0.809, $R^{2}=0.655$), and mean_1st_derivative ($\rho_{S}=-0.699$, r=−0.697, $R^{2}=0.485$). Some metrics are not available in this table (marked “–”), which should be considered when comparing ranks at W=50.

**Table A5:** Correlation summary for α at window length W=50. The first row reports CatBoost predictions against ground truth on scaled windows. All subsequent rows report ground truth vs. metric-derived surrogate values for the three data variants (scaled levels, unscaled levels, returns).

Metric	Scaled Levels			Unscaled Levels			Returns		
	$\rho_{S}$	r	$R^{2}$	$\rho_{S}$	r	$R^{2}$	$\rho_{S}$	r	$R^{2}$
**CatBoost (model)**	**0.934**	**0.932**	**0.869**	–	–	–	–	–	–
hill_index	−0.156	−0.157	0.025	−0.190	−0.190	0.036	−0.269	−0.270	0.073
xi_pickands	0.230	0.230	0.053	0.304	0.303	0.092	0.361	0.361	0.130
xi_moment	−0.153	−0.151	0.023	−0.108	−0.108	0.012	0.182	0.184	0.034
tail_rate	−0.163	−0.163	0.027	−0.164	−0.164	0.027	−0.164	−0.164	0.027
skewness	−0.006	−0.006	0.000	−0.007	−0.007	0.000	0.003	0.003	0.000
kurtosis_excess	0.181	0.183	0.034	0.258	0.258	0.067	0.387	0.389	0.151
hurst_h	–	–	–	–	–	–	–	–	–
hurst_catboost_ml	0.545	0.541	0.293	0.521	0.518	0.268	0.789	0.788	0.621
dfa_alpha	–	–	–	–	–	–	–	–	–
lag1_autocorr	−0.227	−0.002	0.000	−0.227	−0.002	0.000	−0.399	0.002	0.000
ar1_phi	−0.227	−0.002	0.000	−0.227	−0.002	0.000	−0.399	0.002	0.000
theta_from_phi	0.239	0.168	0.028	0.239	0.168	0.028	0.750	0.655	0.428
half_life_from_phi	−0.239	−0.187	0.035	−0.239	−0.187	0.035	−0.750	−0.032	0.001
variance_ratio_q2	−0.413	−0.547	0.299	−0.413	−0.547	0.299	−0.311	−0.493	0.243
variance_ratio_q5	−0.416	−0.548	0.300	−0.416	−0.548	0.300	−0.339	−0.497	0.247
adf_stat	0.334	0.198	0.039	0.350	0.206	0.043	0.262	0.143	0.021
**antropy_sample_entropy**	**−0.897**	**−0.896**	**0.803**	**−0.895**	**−0.894**	**0.799**	**–**	**–**	**–**
**antropy_perm_entropy**	**−0.812**	**−0.809**	**0.655**	**−0.845**	**−0.843**	**0.711**	**−0.745**	**−0.741**	**0.549**
antropy_spectral_entropy	–	–	–	–	–	–	–	–	–
**mean_1st_derivative**	**−0.699**	**−0.697**	**0.485**	**0.059**	**0.060**	**0.004**	**−0.008**	**−0.007**	**0.000**
var_1st_derivative	−0.021	−0.012	0.000	0.124	0.127	0.016	0.078	0.081	0.006
mean_2nd_derivative	−0.669	−0.666	0.443	0.011	0.013	0.000	−0.033	−0.032	0.001
var_2nd_derivative	−0.048	−0.038	0.001	0.093	0.095	0.009	0.053	0.056	0.003
sv_entropy	−0.437	−0.432	0.186	−0.400	−0.397	0.157	−0.247	−0.241	0.058
sv_condition_number	0.446	0.440	0.194	0.407	0.403	0.163	0.240	0.234	0.055
sv_spectral_flatness	−0.447	−0.441	0.195	−0.426	−0.420	0.177	−0.270	−0.262	0.069
sv_effective_rank	−0.437	−0.432	0.186	−0.400	−0.397	0.157	−0.247	−0.241	0.058

At W=250 (Table A6), CatBoost remains strong ($\rho_{S}=0.935$, r=0.934, $R^{2}=0.872$). The entropy metrics strengthen further, especially on returns: antropy_sample_entropy reaches $\rho_{S}=-0.955$ and $R^{2}=0.913$, while antropy_perm_entropy remains substantial (returns: $R^{2}=0.602$). Derivative-based metrics remain strong on scaled levels (e.g., mean_1st_derivative with $R^{2}=0.737$) but continue to be less consistent across preprocessing than the entropy metrics.

At W=365 (Table A7), CatBoost remains strong ($\rho_{S}=0.930$, r=0.930, $R^{2}=0.864$). Entropy metrics remain the most consistent surrogates across all three preprocessing variants. In particular, antropy_sample_entropy is strong on scaled levels ($R^{2}=0.840$) and becomes very strong on returns ($R^{2}=0.919$), with similar behavior for antropy_perm_entropy at lower magnitude. Heavy-tail and tail-related estimators increase with window length (e.g., xi_pickands and kurtosis_excess on returns), but they remain below the entropy-based surrogates in calibration quality and overall explanatory power.

**Table A6:** Correlation summary for α at window length W=250. The first row reports CatBoost predictions against ground truth on scaled windows. All subsequent rows report ground truth vs. metric-derived surrogate values for the three data variants (scaled levels, unscaled levels, returns).The table highlights the results of our model as well as metrics highly correlated with the ground truth in bold.

Metric	Scaled Levels			Unscaled Levels			Returns		
	$\rho_{S}$	r	$R^{2}$	$\rho_{S}$	r	$R^{2}$	$\rho_{S}$	r	$R^{2}$
**CatBoost (model)**	**0.935**	**0.934**	**0.872**	–	–	–	–	–	–
hill_index	−0.476	−0.477	0.228	−0.555	−0.555	0.308	−0.602	−0.602	0.363
xi_pickands	0.488	0.489	0.239	0.586	0.586	0.344	0.699	0.700	0.490
xi_moment	0.159	0.160	0.026	0.228	0.228	0.052	0.579	0.580	0.336
tail_rate	−0.162	−0.162	0.026	−0.162	−0.162	0.026	−0.162	−0.162	0.026
skewness	−0.019	−0.020	0.000	−0.022	−0.022	0.000	−0.020	−0.020	0.000
kurtosis_excess	0.504	0.507	0.257	0.608	0.608	0.369	0.686	0.687	0.472
hurst_h	0.583	0.379	0.144	0.582	0.398	0.159	0.163	0.266	0.071
hurst_catboost_ml	0.471	0.466	0.217	0.501	0.496	0.246	0.820	0.821	0.674
dfa_alpha	−0.314	−0.108	0.012	−0.314	−0.108	0.012	−0.286	−0.214	0.046
lag1_autocorr	−0.051	−0.005	0.000	−0.051	−0.005	0.000	−0.195	0.005	0.000
ar1_phi	−0.051	−0.005	0.000	−0.051	−0.005	0.000	−0.195	0.005	0.000
theta_from_phi	0.052	0.027	0.001	0.052	0.027	0.001	0.680	0.677	0.458
half_life_from_phi	−0.052	−0.045	0.002	−0.052	−0.045	0.002	−0.680	−0.008	0.000
variance_ratio_q2	−0.206	−0.391	0.153	−0.206	−0.391	0.153	−0.177	−0.352	0.124
variance_ratio_q5	−0.202	−0.385	0.148	−0.202	−0.385	0.148	−0.200	−0.348	0.121
adf_stat	0.209	0.101	0.010	0.253	0.129	0.017	0.159	0.076	0.006
**antropy_sample_entropy**	**−0.916**	**−0.915**	**0.838**	**−0.915**	**−0.914**	**0.836**	**−0.955**	**−0.955**	**0.913**
**antropy_perm_entropy**	**−0.870**	**−0.868**	**0.753**	**−0.880**	**−0.878**	**0.771**	**−0.780**	**−0.776**	**0.602**
antropy_spectral_entropy	−0.255	−0.252	0.063	−0.268	−0.264	0.070	0.223	0.231	0.053
**mean_1st_derivative**	**−0.859**	**−0.859**	**0.737**	**0.278**	**0.278**	**0.077**	**0.234**	**0.234**	**0.055**
var_1st_derivative	0.287	0.293	0.086	0.383	0.385	0.149	0.362	0.364	0.132
mean_2nd_derivative	−0.858	−0.858	0.736	0.257	0.257	0.066	0.226	0.226	0.051
var_2nd_derivative	0.257	0.263	0.069	0.363	0.365	0.133	0.345	0.347	0.121
sv_entropy	−0.322	−0.320	0.102	−0.184	−0.182	0.033	−0.069	−0.063	0.004
sv_condition_number	0.321	0.319	0.102	0.185	0.183	0.034	0.053	0.048	0.002
sv_spectral_flatness	−0.324	−0.322	0.103	−0.192	−0.189	0.036	−0.076	−0.070	0.005
sv_effective_rank	−0.322	−0.320	0.102	−0.184	−0.182	0.033	−0.069	−0.063	0.004

**Summary for α across window lengths.** Across Table 7 and Table A5, Table A6 and Table A7, the main conclusion from Section 6.3 remains stable: entropy-based measures are the strongest and most robust classical surrogates for α, with the clearest performance on returns and strong performance on level data. Derivative-based descriptors can be strong on scaled levels but are typically less stable across preprocessing. Heavy-tail estimators and related distributional descriptors can exhibit moderate correlations and improve with larger windows, but they are not competitive with entropy measures as calibrated quantitative surrogates for α in these OU windows. CatBoost maintains consistently high correlations across all window lengths considered.

**Table A7:** Correlation summary for α at window length W=365. The first row reports CatBoost predictions against ground truth on scaled windows. All subsequent rows report ground truth vs. metric-derived surrogate values for the three data variants (scaled levels, unscaled levels, returns). The table highlights the results of our model as well as metrics highly correlated with the ground truth in bold.

Metric	Scaled Levels			Unscaled Levels			Returns		
	$\rho_{S}$	r	$R^{2}$	$\rho_{S}$	r	$R^{2}$	$\rho_{S}$	r	$R^{2}$
**CatBoost (model)**	**0.930**	**0.930**	**0.864**	–	–	–	–	–	–
hill_index	−0.533	−0.534	0.285	−0.617	−0.617	0.381	−0.667	−0.667	0.445
xi_pickands	0.563	0.563	0.317	0.663	0.663	0.439	0.814	0.814	0.662
xi_moment	0.250	0.251	0.063	0.321	0.321	0.103	0.651	0.651	0.424
tail_rate	−0.161	−0.161	0.026	−0.162	−0.162	0.026	−0.162	−0.162	0.026
skewness	−0.025	−0.025	0.001	−0.029	−0.029	0.001	−0.026	−0.026	0.001
kurtosis_excess	0.530	0.533	0.284	0.633	0.633	0.400	0.723	0.724	0.524
hurst_h	0.561	0.385	0.148	0.558	0.400	0.160	0.173	0.268	0.072
hurst_catboost_ml	0.459	0.454	0.206	0.489	0.483	0.234	0.798	0.800	0.640
dfa_alpha	−0.260	−0.156	0.024	−0.260	−0.156	0.024	−0.262	−0.259	0.067
lag1_autocorr	−0.032	0.010	0.000	−0.032	0.010	0.000	−0.153	−0.006	0.000
ar1_phi	−0.032	0.010	0.000	−0.032	0.010	0.000	−0.153	−0.006	0.000
theta_from_phi	0.031	0.019	0.000	0.031	0.019	0.000	0.639	0.672	0.452
half_life_from_phi	−0.031	−0.140	0.020	−0.031	−0.140	0.020	−0.639	−0.097	0.009
variance_ratio_q2	−0.164	−0.343	0.118	−0.164	−0.343	0.118	−0.150	−0.311	0.096
variance_ratio_q5	−0.158	−0.336	0.113	−0.158	−0.336	0.113	−0.166	−0.306	0.094
adf_stat	0.168	0.090	0.008	0.219	0.102	0.010	0.179	0.065	0.004
**antropy_sample_entropy**	**−0.917**	**−0.917**	**0.840**	**−0.917**	**−0.916**	**0.840**	**−0.959**	**−0.959**	**0.919**
**antropy_perm_entropy**	**−0.879**	**−0.877**	**0.769**	**−0.884**	**−0.882**	**0.778**	**−0.783**	**−0.779**	**0.607**
antropy_spectral_entropy	−0.242	−0.239	0.057	−0.254	−0.251	0.063	0.257	0.265	0.070
**mean_1st_derivative**	**−0.874**	**−0.875**	**0.765**	**0.329**	**0.329**	**0.108**	**0.289**	**0.289**	**0.083**
var_1st_derivative	0.339	0.343	0.118	0.435	0.436	0.190	0.418	0.420	0.176
mean_2nd_derivative	−0.877	−0.876	0.768	0.312	0.313	0.098	0.285	0.285	0.081
var_2nd_derivative	0.271	0.275	0.076	0.419	0.420	0.177	0.405	0.407	0.165
sv_entropy	−0.297	−0.296	0.088	−0.138	−0.136	0.018	−0.030	−0.025	0.001
sv_condition_number	0.295	0.294	0.086	0.140	0.138	0.019	0.012	0.008	0.000
sv_spectral_flatness	−0.298	−0.297	0.088	−0.144	−0.143	0.020	−0.034	−0.030	0.001
sv_effective_rank	−0.297	−0.296	0.088	−0.138	−0.136	0.018	−0.030	−0.025	0.001

### Appendix C.2. Additional Results for θ

Table A8, Table A9 and Table A10 report the correlation summaries for θ at W∈{50, 250, 365}. These results complement Table 6 in Section 6.3 and allow a comparison of the OU-consistent proxy metrics and alternative descriptors across window lengths.

At W=50 (Table A8), CatBoost shows weaker performance than for larger windows ($\rho_{S}=0.810$, r=0.843, $R^{2}=0.710$), consistent with reduced information in shorter windows. Among classical metrics on scaled levels, theta_from_phi remains the strongest OU-consistent proxy ($\rho_{S}=0.694$, r=0.713, $R^{2}=0.509$). Kurtosis and the learned Hurst surrogate hurst_catboost_ml provide moderate performance on levels, while many other metrics are weakly calibrated. On unscaled levels, SVD-based metrics (e.g., sv_entropy and sv_effective_rank) become relatively stronger than on scaled levels (e.g., $R^{2}$ up to 0.553), indicating sensitivity of these descriptors to amplitude structure that is removed by scaling. On returns, most θ surrogates remain weak, including theta_from_phi, consistent with the loss of level-based mean-reversion information under differencing.

**Table A8:** Correlation summary for θ at window length W=50. The first row reports CatBoost predictions against ground truth on scaled windows. All subsequent rows report ground truth vs. metric-derived surrogate values for the three data variants (scaled levels, unscaled levels, returns). The table highlights the results of our model as well as metrics highly correlated with the ground truth in bold.

Metric	Scaled Levels			Unscaled Levels			Returns		
	$\rho_{S}$	r	$R^{2}$	$\rho_{S}$	r	$R^{2}$	$\rho_{S}$	r	$R^{2}$
**CatBoost (model)**	**0.810**	**0.843**	**0.710**	–	–	–	–	–	–
hill_index	0.617	0.010	0.000	0.630	0.011	0.000	0.170	0.010	0.000
xi_pickands	0.364	0.217	0.047	0.328	0.210	0.044	0.054	0.065	0.004
xi_moment	0.347	0.242	0.059	0.314	0.208	0.043	0.046	0.036	0.001
tail_rate	0.039	0.029	0.001	0.273	0.177	0.031	0.273	0.177	0.031
skewness	0.003	0.001	0.000	0.002	0.001	0.000	0.000	0.000	0.000
**kurtosis_excess**	**0.601**	**0.566**	**0.320**	**0.585**	**0.567**	**0.321**	0.052	0.007	0.000
hurst_h	–	–	–	–	–	–	–	–	–
**hurst_catboost_ml**	**0.542**	**0.502**	**0.252**	**0.501**	**0.484**	**0.235**	0.113	0.115	0.013
dfa_alpha	–	–	–	–	–	–	–	–	–
lag1_autocorr	0.000	0.003	0.000	0.001	0.003	0.000	0.003	0.003	0.000
ar1_phi	0.000	0.003	0.000	0.001	0.003	0.000	0.003	0.003	0.000
**theta_from_phi**	**0.694**	**0.713**	**0.509**	**0.694**	**0.713**	**0.509**	−0.038	0.078	0.006
half_life_from_phi	0.694	0.152	0.023	0.694	0.152	0.023	0.047	0.026	0.001
variance_ratio_q2	0.291	0.215	0.046	0.291	0.215	0.046	0.044	0.034	0.001
variance_ratio_q5	0.352	0.258	0.066	0.351	0.258	0.066	0.083	0.077	0.006
adf_stat	0.256	0.064	0.004	0.256	0.102	0.010	0.107	0.140	0.020
antropy_sample_entropy	0.133	0.159	0.025	0.170	0.164	0.027	–	–	–
antropy_perm_entropy	0.001	0.081	0.007	0.091	0.143	0.021	0.025	0.103	0.011
antropy_spectral_entropy	–	–	–	–	–	–	–	–	–
mean_1st_derivative	0.302	0.298	0.089	0.019	0.006	0.000	0.018	0.006	0.000
var_1st_derivative	0.212	0.105	0.011	0.010	0.005	0.000	0.009	0.005	0.000
mean_2nd_derivative	0.271	0.280	0.078	0.018	0.006	0.000	0.018	0.006	0.000
var_2nd_derivative	0.259	0.254	0.065	0.009	0.005	0.000	0.009	0.005	0.000
sv_entropy	0.274	0.363	0.132	**0.659**	**0.724**	**0.525**	−0.142	0.067	0.004
sv_condition_number	0.002	0.003	0.000	0.002	0.003	0.000	0.002	0.003	0.000
sv_spectral_flatness	0.276	0.358	0.128	0.645	0.707	0.499	−0.138	0.070	0.005
sv_effective_rank	0.274	0.366	0.134	**0.659**	**0.743**	**0.553**	−0.142	0.061	0.004

At W=250 (Table A9), both CatBoost and theta_from_phi become very strong and closely matched: CatBoost achieves $\rho_{S}=0.960$ and $R^{2}=0.942$, while theta_from_phi achieves $\rho_{S}=0.944$ and $R^{2}=0.943$ on scaled levels (and identical values on unscaled levels). Spectral entropy also remains informative ($R^{2}=0.499$ on scaled levels), while kurtosis remains moderate ($R^{2}=0.427$). As in the main section, returns remain comparatively weak for θ-related surrogates (e.g., theta_from_phi with $R^{2}=0.049$), confirming that the most informative structure for θ is carried by level dynamics rather than differenced dynamics in this analysis.

At W=365 (Table A10), CatBoost is strongest ($\rho_{S}=0.973$, r=0.975, $R^{2}=0.951$), and theta_from_phi becomes an even stronger surrogate on levels ($\rho_{S}=0.964$, r=0.985, $R^{2}=0.970$). Spectral entropy remains a stable secondary descriptor (scaled levels: $R^{2}=0.513$), while kurtosis remains moderate ($R^{2}=0.427$). As in other window sizes, returns are weak for the OU-consistent proxy and for kurtosis, and only modest for spectral entropy.

**Table A9:** Correlation summary for θ at window length W=250. The first row reports CatBoost predictions against ground truth on scaled windows. All subsequent rows report ground truth vs. metric-derived surrogate values for the three data variants (scaled levels, unscaled levels, returns). The table highlights the results of our model as well as metrics highly correlated with the ground truth in bold.

Metric	Scaled Levels			Unscaled Levels			Returns		
	$\rho_{S}$	r	$R^{2}$	$\rho_{S}$	r	$R^{2}$	$\rho_{S}$	r	$R^{2}$
**CatBoost (model)**	**0.960**	**0.971**	**0.942**	–	–	–	–	–	–
hill_index	0.603	0.013	0.000	0.625	0.003	0.000	0.326	0.004	0.000
xi_pickands	0.442	0.295	0.087	0.413	0.291	0.085	0.221	0.225	0.050
xi_moment	0.647	0.483	0.233	0.642	0.411	0.169	0.183	0.156	0.024
tail_rate	0.070	0.045	0.002	0.272	0.175	0.031	0.272	0.175	0.031
skewness	0.016	0.013	0.000	0.015	0.012	0.000	0.002	0.002	0.000
**kurtosis_excess**	**0.653**	**0.654**	**0.427**	**0.637**	**0.653**	**0.426**	**−0.002**	**0.009**	**0.000**
hurst_h	−0.163	0.147	0.022	−0.179	0.130	0.017	−0.126	0.036	0.001
hurst_catboost_ml	0.434	0.393	0.155	0.460	0.415	0.172	0.183	0.188	0.035
dfa_alpha	0.541	0.302	0.091	0.541	0.302	0.091	0.283	0.146	0.021
lag1_autocorr	0.943	0.004	0.000	−0.006	0.006	0.000	−0.001	0.003	0.000
ar1_phi	0.943	0.004	0.000	−0.006	0.006	0.000	−0.001	0.003	0.000
**theta_from_phi**	**0.944**	**0.971**	**0.943**	**0.944**	**0.971**	**0.943**	**0.134**	**0.221**	**0.049**
half_life_from_phi	0.944	0.100	0.010	0.944	0.100	0.010	−0.127	0.005	0.000
variance_ratio_q2	0.634	0.288	0.083	0.634	0.288	0.083	0.229	0.081	0.007
variance_ratio_q5	0.710	0.301	0.090	0.710	0.301	0.090	0.377	0.099	0.010
adf_stat	−0.009	0.018	0.000	−0.026	0.034	0.001	−0.246	0.070	0.005
antropy_sample_entropy	0.199	0.275	0.076	0.222	0.276	0.076	0.043	0.002	0.000
antropy_perm_entropy	0.006	0.096	0.009	0.051	0.136	0.019	−0.048	0.107	0.011
**antropy_spectral_entropy**	**0.735**	**0.706**	**0.499**	**0.745**	**0.694**	**0.482**	**0.104**	**0.188**	**0.036**
mean_1st_derivative	0.246	0.291	0.085	0.033	0.013	0.000	0.033	0.013	0.000
var_1st_derivative	0.258	0.242	0.058	0.017	0.011	0.000	0.017	0.011	0.000
mean_2nd_derivative	0.236	0.304	0.092	0.033	0.013	0.000	0.033	0.013	0.000
var_2nd_derivative	0.420	0.421	0.177	0.017	0.011	0.000	0.017	0.011	0.000
sv_entropy	0.348	0.387	0.149	0.872	0.903	0.815	−0.502	0.093	0.009
sv_condition_number	0.004	0.002	0.000	0.028	0.004	0.000	0.012	0.004	0.000
sv_spectral_flatness	0.357	0.398	0.158	0.868	0.893	0.798	−0.501	0.091	0.008
sv_effective_rank	0.348	0.380	0.144	0.860	0.919	0.844	−0.502	0.082	0.007

**Summary for θ across window lengths.** Across Table 6 and Table A8, Table A9 and Table A10, the main conclusion from Section 6.3 remains stable: θ is best captured by an OU-consistent proxy derived from an AR(1) structure, specifically theta_from_phi, which becomes increasingly well calibrated as window length grows. Spectral entropy and excess kurtosis provide additional but weaker information on level data, while many heavy-tail and generic distributional metrics show limited calibration for θ in this setting. Across all window lengths, differencing into returns substantially reduces the strength of θ surrogates, consistent with the loss of a level-based mean-reversion structure. CatBoost benefits from longer windows and remains consistently strong across the range of window lengths reported here.

**Table A10:** Correlation summary for θ at window length W=365. The first row reports CatBoost predictions against ground truth on scaled windows. All subsequent rows report ground truth vs. metric-derived surrogate values for the three data variants (scaled levels, unscaled levels, returns). The table highlights the results of our model as well as metrics highly correlated with the ground truth in bold.

Metric	Scaled Levels			Unscaled Levels			Returns		
	$\rho_{S}$	r	$R^{2}$	$\rho_{S}$	r	$R^{2}$	$\rho_{S}$	r	$R^{2}$
**CatBoost (model)**	**0.973**	**0.975**	**0.951**	–	–	–	–	–	–
hill_index	0.600	0.016	0.000	0.594	0.003	0.000	0.325	0.003	0.000
xi_pickands	0.410	0.271	0.073	0.382	0.257	0.066	0.188	0.202	0.041
xi_moment	0.669	0.489	0.239	0.667	0.425	0.181	0.211	0.214	0.046
tail_rate	0.088	0.055	0.003	0.272	0.175	0.031	0.272	0.175	0.031
skewness	0.020	0.015	0.000	0.019	0.014	0.000	0.003	0.003	0.000
**kurtosis_excess**	**0.650**	**0.654**	**0.427**	**0.634**	**0.651**	**0.424**	**0.008**	**0.020**	**0.000**
hurst_h	−0.144	0.164	0.027	−0.166	0.144	0.021	−0.106	0.059	0.003
hurst_catboost_ml	0.379	0.334	0.111	0.407	0.357	0.127	0.195	0.189	0.036
dfa_alpha	0.647	0.452	0.205	0.647	0.452	0.205	0.370	0.195	0.038
lag1_autocorr	0.964	0.011	0.000	0.964	0.011	0.000	−0.709	0.009	0.000
ar1_phi	0.964	0.011	0.000	0.964	0.011	0.000	−0.709	0.009	0.000
**theta_from_phi**	**0.964**	**0.985**	**0.970**	**0.964**	**0.985**	**0.970**	**0.191**	**0.272**	**0.074**
half_life_from_phi	0.964	0.101	0.010	0.964	0.101	0.010	−0.192	0.078	0.006
variance_ratio_q2	0.702	0.311	0.097	0.702	0.311	0.097	0.282	0.080	0.006
variance_ratio_q5	0.769	0.316	0.100	0.769	0.316	0.100	0.454	0.092	0.008
adf_stat	−0.027	0.005	0.000	−0.028	0.028	0.001	−0.404	0.054	0.003
antropy_sample_entropy	0.199	0.285	0.081	0.217	0.285	0.081	0.029	0.000	0.000
antropy_perm_entropy	0.007	0.091	0.008	0.037	0.128	0.016	−0.071	0.101	0.010
**antropy_spectral_entropy**	**0.744**	**0.716**	**0.513**	**0.750**	**0.702**	**0.493**	**0.087**	**0.174**	**0.030**
mean_1st_derivative	0.235	0.279	0.078	0.039	0.017	0.000	0.039	0.017	0.000
var_1st_derivative	0.271	0.277	0.077	0.021	0.014	0.000	0.021	0.014	0.000
mean_2nd_derivative	0.221	0.292	0.085	0.039	0.017	0.000	0.039	0.017	0.000
var_2nd_derivative	0.466	0.464	0.215	0.021	0.014	0.000	0.021	0.014	0.000
sv_entropy	0.343	0.371	0.137	0.902	0.925	0.856	−0.598	0.081	0.007
sv_condition_number	−0.026	0.003	0.000	0.031	0.005	0.000	0.012	0.004	0.000
sv_spectral_flatness	0.355	0.382	0.146	0.899	0.917	0.842	−0.598	0.079	0.006
sv_effective_rank	0.343	0.364	0.133	0.898	0.940	0.883	−0.598	0.069	0.005

## Appendix D. Additional Results Financial Time-Series Data

Table A11 and Table A12 report absolute counts per interval and asset for the binary versions of α (Gaussian vs. Lévy) and θ (mean reversion vs. no mean reversion). Counts equal the number of rolling windows falling into each binary class within the given period.

**Table A11:** Absolute binary counts for α (Gaussian vs. Lévy) by period and asset.

Period	Asset	Gaussian	Lévy	Rows
1995–2010	AAPL	2297	1481	3778
	DJI	2954	824	3778
	GSPC	2982	796	3778
	MSFT	2560	1218	3778
2010–2024	AAPL	2325	1448	3773
	DJI	2472	1301	3773
	GSPC	2451	1322	3773
	MSFT	2518	1255	3773

**Table A12:** Absolute binary counts for θ (mean reversion vs. no mean reversion) by period and asset.

Period	Asset	Mean Reversion	No Mean Reversion	Rows
1995–2010	AAPL	2290	1488	3778
	DJI	2597	1181	3778
	GSPC	2584	1194	3778
	MSFT	2349	1429	3778
2010–2024	AAPL	2412	1361	3773
	DJI	2540	1233	3773
	GSPC	2492	1281	3773
	MSFT	2528	1245	3773

## Appendix E. Additional NASA Power Data

**Table A13:** Binary α counts by period and variable (all Austrian locations pooled; 50-day windows, step 1).

Period	Variable	Gaussian	Lévy	Rows
1985–2004	ALLSKY_SFC_SW_DWN	252,661	273,299	525,960
	CLRSKY_SFC_SW_DWN	310,660	215,300	525,960
	PRECTOTCORR	368	525,592	525,960
	PS	442,210	83,750	525,960
	RH2M	309,072	216,888	525,960
	T2MDEW	419,656	106,304	525,960
	T2M_RANGE	354,956	171,004	525,960
	TOA_SW_DWN	0	525,960	525,960
	WS10M	230,010	295,950	525,960
	WS10M_MAX	197,802	328,158	525,960
2005–2025	ALLSKY_SFC_SW_DWN	267,713	258,247	525,960
	CLRSKY_SFC_SW_DWN	395,689	130,271	525,960
	PRECTOTCORR	133	525,827	525,960
	PS	431,877	94,083	525,960
	RH2M	307,729	218,231	525,960
	T2MDEW	407,164	118,796	525,960
	T2M_RANGE	355,124	170,836	525,960
	TOA_SW_DWN	0	525,960	525,960
	WS10M	218,228	307,732	525,960
	WS10M_MAX	179,446	346,514	525,960

**Table A14:** Binary θ counts by period and variable (all Austrian locations pooled; 50-day windows, step 1).

Period	Variable	no_mean_rev	mean_rev	Rows
1985–2004	ALLSKY_SFC_SW_DWN	8878	517,082	525,960
	CLRSKY_SFC_SW_DWN	158,849	367,111	525,960
	PRECTOTCORR	35,351	490,609	525,960
	PS	29,122	496,838	525,960
	RH2M	15,784	510,176	525,960
	T2MDEW	62,139	463,821	525,960
	T2M_RANGE	719	525,241	525,960
	TOA_SW_DWN	302,176	223,784	525,960
	WS10M	2009	523,951	525,960
	WS10M_MAX	2624	523,336	525,960
2005–2025	ALLSKY_SFC_SW_DWN	11,134	514,826	525,960
	CLRSKY_SFC_SW_DWN	188,429	337,531	525,960
	PRECTOTCORR	34,683	491,277	525,960
	PS	30,259	495,701	525,960
	RH2M	14,769	511,191	525,960
	T2MDEW	67,102	458,858	525,960
	T2M_RANGE	941	525,019	525,960
	TOA_SW_DWN	302,471	223,489	525,960
	WS10M	1903	524,057	525,960
	WS10M_MAX	2185	523,775	525,960
